# Delivery of CDNF by AAV-mediated gene transfer protects dopamine neurons and regulates ER stress and inflammation in an acute MPTP mouse model of Parkinson’s disease

**DOI:** 10.1038/s41598-024-65735-5

**Published:** 2024-07-17

**Authors:** Jinhan Nam, Christopher T. Richie, Brandon K. Harvey, Merja H. Voutilainen

**Affiliations:** 1 Division of Pharmacology and Pharmacotherapy, Faculty of Pharmacy, University of Helsinki, Viikinkaari 5E, P.O. Box 56, 00014 Helsinki, Finland; 2 Intramural Research Program, National Institute on Drug Abuse, Baltimore, MD USA

**Keywords:** Parkinson’s disease, 1-Methyl-4-phenyl-1,2,3,6-tetrahydropyridine, Cerebral dopamine neurotrophic factor, Neuroinflammation, Unfolded protein response, Microglia, Astroglia, Pharmacology, Parkinson's disease

## Abstract

Cerebral dopamine neurotrophic factor (CDNF) and its close structural relative, mesencephalic astrocyte-derived neurotrophic factor (MANF), are proteins with neurotrophic properties. CDNF protects and restores the function of dopamine (DA) neurons in rodent and non-human primate (NHP) toxin models of Parkinson’s disease (PD) and therefore shows promise as a drug candidate for disease-modifying treatment of PD. Moreover, CDNF was found to be safe and to have some therapeutic effects on PD patients in phase 1/2 clinical trials. However, the mechanism underlying the neurotrophic activity of CDNF is unknown. In this study, we delivered human CDNF (hCDNF) to the brain using an adeno-associated viral (AAV) vector and demonstrated the neurotrophic effect of AAV-hCDNF in an acute 1-methyl-4-phenyl-1,2,3,6-tetrahydropyridine (MPTP) mouse model of PD. AAV-hCDNF resulted in the expression of hCDNF in the striatum (STR) and substantia nigra (SN), and no toxic effects on the nigrostriatal pathway were observed. Intrastriatal injection of AAV-hCDNF reduced motor impairment and partially alleviated gait dysfunction in the acute MPTP mouse model. In addition, gene therapy with AAV-hCDNF had significant neuroprotective effects on the nigrostriatal pathway and decreased the levels of interleukin 1beta (IL-1β) and complement 3 (C3) in glial cells in the acute MPTP mouse model. Moreover, AAV-hCDNF reduced C/EBP homologous protein (CHOP) and glucose regulatory protein 78 (GRP78) expression in astroglia. These results suggest that the neuroprotective effects of CDNF may be mediated at least in part through the regulation of neuroinflammation and the UPR pathway in a mouse MPTP model of PD in vivo.

## Introduction

Cerebral dopamine neurotrophic factor (CDNF) was first described in 2007^1^. Since then, the roles of CDNF under both physiological and pathological conditions have been studied extensively. When recombinant human CDNF (rhCDNF) is injected into the striatum (STR), it is endocytosed and retrogradely transported from the striatum (STR) to the substantia nigra (SN) in the brain^2–4^. Moreover, a study using CDNF knockout mice showed that CDNF deficiency leads to the degeneration of enteric neurons and constipation, which are also early symptoms of Parkinson’s disease (PD)^5,6^. Furthermore, the expression of unfolded protein response (UPR) pathway genes in the skeletal muscle was found to be elevated in CDNF knockout mice compared with wild-type mice^7^. Delivery of CDNF gene using an adeno-associated viral (AAV) vector (AAV2-CDNF) was shown to improve long-term memory in APP/PS1 transgenic mice^8^. rhCDNF protein was found to prevent neuronal death in a quinolinic acid model of Huntington’s disease (HD)^9^ and to improve motor coordination and protect motor neurons from degeneration through regulation of the UPR pathway in three different genetic rodent models of amyotrophic lateral sclerosis (ALS)^10^. Collectively, these findings indicate that CDNF has neurotrophic effects in various neurodegenerative diseases.

PD is a neurodegenerative disease characterized by progressive degeneration of the nigrostriatal dopaminergic pathway, causing several motor symptoms, such as rigidity, bradykinesia, resting tremor, and gait disturbance, and non-motor symptoms, including gastrointestinal dysfunctions, sleep disorders, and depression^11,12^. The neurotrophic effects of the CDNF protein were first investigated in a 6-hydroxydopamine (6-OHDA) rat model of PD^1^. Thereafter, the neuroprotective and neurorestorative effects of CDNF have been tested in various rodent models of PD, such as in the 1-methyl-4-phenyl-1,2,3,6-tetrahydropyridine (MPTP), and α-synuclein preformed fibril (PFF)-induced PD models^1,3,13–18^. However, the aim of most of these studies was to find ways to improve motor functions and stop the degeneration of the nigrostriatal dopamine pathway. The exact mechanisms by which CDNF rescues these symptoms are still unknown.

Neuroinflammation is one of the cellular hallmarks of various neurodegenerative diseases; it is mediated by the innate immune system of the central nervous system (CNS) and controlled by glial cells, including astroglia and microglia^19–22^. Microglia and astroglia have important functions in the CNS under mild neuroinflammatory conditions, such as homeostasis of neurotransmitters, regulation of the immune response, secretion of anti-inflammatory cytokines and secretion of growth factors. Neuroinflammation is a defence response for preventing neuronal death^23^. The overall cascade of neuroinflammation involves a variety of signalling molecules, including reactive oxygen species (ROS), reactive nitrogen species (RNS), cytokines, and chemokines. For instance, in chronic conditions, microglia and astroglia are activated by ROS/RNS produced by diseased neurons, and in response, glial cells secrete proinflammatory cytokines such as interleukin-1 beta (IL-1β), complement 3 (C3), tumour necrosis factor-alpha (TNF-α), interleukin-6 (IL-6), and interferon-gamma (IFN-γ)^20,24–26^. These proinflammatory mediators can act on neurons to promote neuronal death. This process can affect other healthy cells and promote the transition from a healthy to a harmful cellular environment within the CNS. Accumulating data have established that reactive microglia and astroglia play a critical role in the initiation and development of cell death in many neurodegenerative disorders, including PD^27–29^.

The UPR is an adaptive response that helps maintain proteostasis in the cell by regulating the folding, translation, and degradation of proteins in the endoplasmic reticulum (ER). The UPR is mediated through three distinct signal transduction pathways that are regulated by glucose regulatory protein 78 (GRP78): the protein kinase RNA (PKR)-like ER kinase (PERK), inositol-requiring protein 1 alpha (IRE1α), and activating transcription factor 6 (ATF6) pathways. Initially, the UPR has a protective effect. However, during chronic ER stress, the UPR stimulates apoptotic pathways^30,31^. A growing body of evidence indicates the role of chronic ER stress in the development of PD, both in human patients and animal models^32–35^.

In the present study, we delivered human CDNF (hCDNF) to an acute MPTP mouse model of PD via an AAV vector and found that prolonged expression of hCDNF attenuated the degeneration of the nigrostriatal pathway and the associated deficits in motor function and gait. We also found that hCDNF expression reduced neuroinflammation and ER stress in glial cells in an acute MPTP mouse model of PD.

## Results

### Characterization of the expression and toxicity of AAV-GFP and AAV-hCDNF after injection into the striata of mice

**Figure 1:**
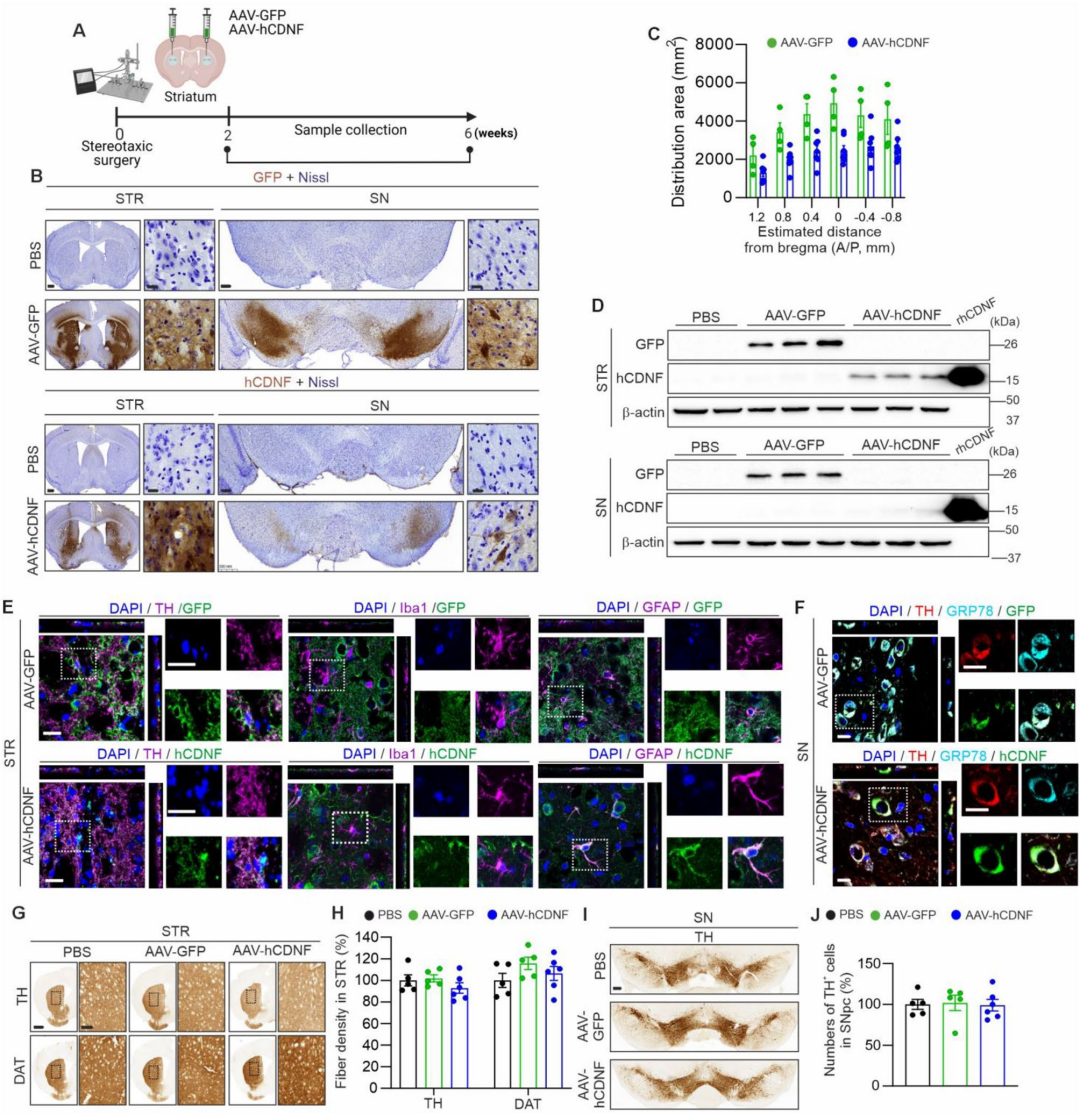
Characterization of the effects of the intrastriatal delivery of AAV-GFP and AAV-hCDNF at 6 weeks. (**A**) Experimental scheme for analysing the transduction of AAV-GFP and AAV-hCDNF in the mouse brain. C57BL/6 mice received bilateral intrastriatal injections of AAV-GFP, AAV-hCDNF, or PBS as a control. Samples were collected at 2, 4 and 6 weeks after injection. (**B**) Immunohistochemical staining of striatum (STR) and substantia nigra (SN) sections for GFP (upper panel, brown) and CDNF (lower panel, brown) and counterstaining with cresyl violet (Nissl bodies). Scale bars: 500 µm for the STR, 200 µm for the SN, and 20 µm for the enlarged images. (**C**) Distribution profiles of GFP and hCDNF at 6 weeks after injection of AAV-GFP or AAV-hCDNF into the STR. (**D**) Western blot analysis of GFP and CDNF expression in the STR and SN. (**E**) Immunofluorescence staining of GFP (green) or hCDNF (green) in dopaminergic neurons (TH; magenta), microglia (Iba1; magenta) and astroglia (GFAP: magenta) with in the STR. Scale bar, 20 µm. (**F**) Double immunofluorescence staining of nuclei (DAPI, cyan) and GRP78 (red) with GFP (green) or hCDNF (green) in the SNpc. Scale bar, 20 µm. (**G**) Immunohistochemical staining of striatal sections for TH (upper) and DAT (lower). Scale bar, 1 mm, 200 μm for enlarged images of the STR. (**H**) Quantification of the TH^+^ or DAT^+^ fibre density in the STR. (**I**) Immunohistochemical staining of SN sections for TH. Scale bar, 200 µm. (**J**) Quantification of TH^+^ neurons in the SNpc. (**C**) *n* = 4 for the AAV-GFP group,* n* = 7 for the AAV-hCDNF group, (**H**,**J**) *n* = 5 for the PBS group,* n* = 5 for the AAV-GFP group, and *n* = 6 for the AAV-hCDNF group. (**H**,**J**) One-way ANOVA followed by Tukey’s multiple comparisons post hoc test. All the data are expressed as the mean ± S.E.M.

To investigate the effects of hCDNF in the MPTP mouse model of PD, we injected an AAV vector carrying hCDNF or GFP (as a control) or PBS into the striata of mice. The experimental timeline is shown in Fig. 1A, and the results from 6 weeks after AAV injection are shown in Fig. 1. The mice were injected bilaterally with AAV-GFP, AAV-hCDNF, or PBS into the striatum, and the brains were collected after 2 or 6 weeks. The expression of GFP and hCDNF was visualized by immunostaining with DAB, and the tissues were counterstained with cresyl violet to visualize Nissl bodies. GFP and hCDNF were expressed in the STR (Fig. 1B,C) and in the SNpc at 2 and 6 weeks, but the expression of CDNF in the SNpc was low relative to that of GFP (Fig. 1B, Supplementary Fig. 1A). Western blot analysis corroborated the immunostaining data, showing that the transgene proteins were expressed in the STR (Fig. 1D). To determine the distribution of GFP- and hCDNF-transduced cells in the STR and SNpc, tissues were subjected to double immunofluorescence staining for tyrosine hydroxylase (TH) to label dopaminergic fibres in the STR and dopaminergic neurons in the SN, ionized calcium binding adaptor molecule 1 (Iba1) to label microglia, or glial fibrillary acidic protein (GFAP) to label astroglia and GFP or CDNF. hCDNF expression was detected in TH-positive fibres, in GFAP-positive cells in the STR, and in dopamine neurons in the SN but not in Iba1-positive cells in the STR and SN (Fig. 1E,F, Supplementary Fig. 1B). To study whether hCDNF and GFP were localized in the lumen of the ER, we performed double immunofluorescence staining for GFP or hCDNF together with GRP78, a resident protein of the ER lumen, the SN. The GFP and hCDNF signals colocalized with the GRP78 signal (Fig. 1F). hCDNF has a Lys-Thr-Glu-Leu or “KTEL” amino acid sequence in the C-terminus, which acts as an ER retention signal sequence^36^. The GFP used as a control was modified to have the same KTEL sequence at its C-terminus; thus, it should have also been localized to the ER.

To test the potential toxicity of AAV-mediated transgene protein expression to dopaminergic neurons in the striatum, we examined dopaminergic fibres and neurons in the STR and SN by immunostaining for TH and DAT. At 6 weeks after intrastriatal injection, compared with PBS, neither AAV-hCDNF nor AAV-GFP significantly changed TH and DAT immunoreactivity in the striatum (Fig. 1G,H) or the number of TH-positive dopamine neurons in the SNpc (Fig. 1I,J). Similar results were observed at 2 weeks after injection (Supplementary Fig. 1C,D). These results suggest that hCDNF and GFP-KTEL were expressed in the ER of cells in the STR and SN and that their expression did not cause overt toxicity to dopaminergic neurons or their processes.

### Treatment with hCDNF mitigates MPTP-induced impairment of motor function but not gait dynamics

**Figure 2:**
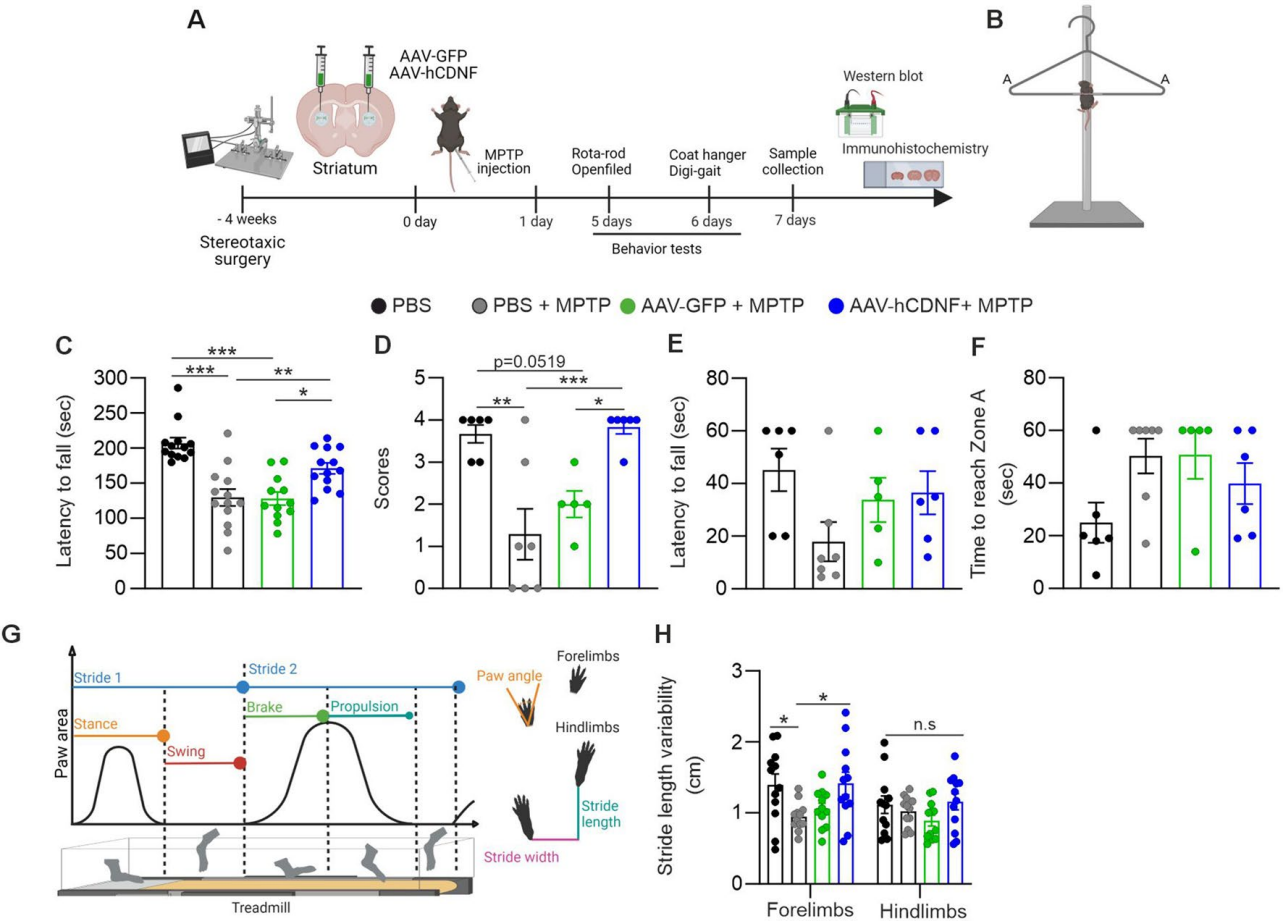
hCDNF alleviates motor function impairments and improves gait dynamics in an acute MPTP mouse model of PD. (**A**) Diagram of the experimental design. C57BL/6 mice received bilateral intrastriatal injections of AAV-GFP, AAV-hCDNF, or PBS. Four weeks later, the mice were intraperitoneally injected with PBS or MPTP four times per day at 2 h intervals. The mice were sacrificed 7 days after the last MPTP injection. Before the mice were sacrificed, behavioural tests (the rotarod test, coat hanger test, and open field test and DigiGait analysis) were performed at 5 and 6 days after MPTP injection. (**B**) Illustration of the coat hanger test. (**C**) The latency to fall off the rotarod was recorded. (**D**) Evaluation of motor score in the coat hanger test. (**E**) The latency to fall off the coat hanger was recorded. (**F**) The time to reach zone A in the coat hanger test was recorded. (**G**) Schematic diagram of gait data (left) and paw print data (right) from DigiGait analysis. The gait of each mouse on a transparent treadmill was recorded by a digital camera while the mouse walked. One stride consisted of a stance phase (the time during which the paw contacted the belt) and swing phase (the time during which the paw did not contact the belt). One stance phase had two subphases: brake and propulsion. Brake was the first subphase of the stance phase and was defined as the initial paw contact to maximum paw contact after commencing the swing phase. Propulsion was defined as the duration of maximum paw contact just before the swing phase. Additionally, stride length, width and paw angle were analysed from the paw print data. (**H**) Stride length variability. (**C**) *n* = 13 for the PBS group, *n* = 13 for the PBS + MPTP group, *n* = 12 for the AAV-GFP + MPTP; AAV-GFP + MPTP group, and *n* = 13 for the AAV-hCDNF + MPTP group. (**D**–**F**) *n* = 6 for the PBS group, *n* = 7 for the PBS + MPTP group, *n* = 5 for the AAV-GFP + MPTP group, and *n* = 6 for the AAV-hCDNF + MPTP group. (**H**) *n* = 12 for the PBS group, *n* = 12 for the PBS + MPTP group, *n* = 12 for the AAV-GFP + MPTP group, and *n* = 12 for the AAV-hCDNF + MPTP group. One-way ANOVA followed by Tukey’s multiple comparisons post hoc test. All the data are expressed as the mean ± S.E.M. *p < 0.05, **p < 0.01, and ***p < 0.001. n.s.: not significant.

MPTP-induced lesioning of the nigrostriatal pathway in mice is a method for modeling acute PD that has been widely used for evaluating potential therapeutics for the treatment of PD^18,37^. Here, we used the MPTP model to test the ability of AAV-hCDNF to alleviate behavioural changes associated with the loss of dopaminergic neurons (Fig. 2A). To investigate the effects of AAV-hCDNF on motor function, we performed behavioural tests, such as rotarod test, coat hanger test, locomotor activity test, and DigiGait analysis. These behavioural tests are widely used to evaluate motor coordination and functional deficits in rodent models of PD. In the rotarod test, MPTP impaired motor coordination, whereas AAV-hCDNF improved motor coordination in MPTP-treated mice (Fig. 2C). In the coat hanger test (Fig. 2B), the scores of the PBS + MPTP- and AAV-GFP + MPTP-treated mice were lower than those of the PBS-treated mice, and the scores of the AAV-hCDNF + MPTP-treated mice were significantly greater. In other words, improvements in motor function in the AAV-hCDNF group were detected in the coat hanger test, and compared with PBS + MPTP and AAV-GFP + MPTP treatment, AAV-hCDNF treatment significantly increased forelimb strength and motor coordination (Fig. 2D). There was no significant difference in hanging time (Fig. 2E) or the time to reach the edge of the diagonal bar (Fig. 2F). We assessed locomotor activity by measuring the distance travelled and number of vertical movements (rearings), which have been shown to be metrics for evaluating locomotor impairment in PD models^38^. There was no difference in the distance travelled or number of vertical movements between the experimental groups (Supplementary Fig. 2).

The presence of gait impairment, or difficulties in walking, is used as an indicator for the clinical diagnosis of PD in patients^39–41^. Thus, we investigated whether exogenous hCDNF mitigates MPTP-induced changes in gait and paw placement (Fig. 2G). A variety of gait parameters were analysed from videos (Supplementary Table 1). When compared with PBS treatment, acute MPTP treatment significantly decreased forelimb stride length variability. Moreover, MPTP-induced gait impairments were alleviated by AAV-hCDNF (Fig. 2H). Analysis of other gait dynamics parameters revealed no differences between the acute MPTP model mice and the control, AAV-GFP or AAV-hCDNF group (Supplementary Fig. S3). Collectively, these results indicate that AAV-hCDNF ameliorated motor deficits caused by MPTP but not changes in gait dynamics.

### hCDNF prevents MPTP-induced degeneration of the nigrostriatal dopamine pathway

**Figure 3:**
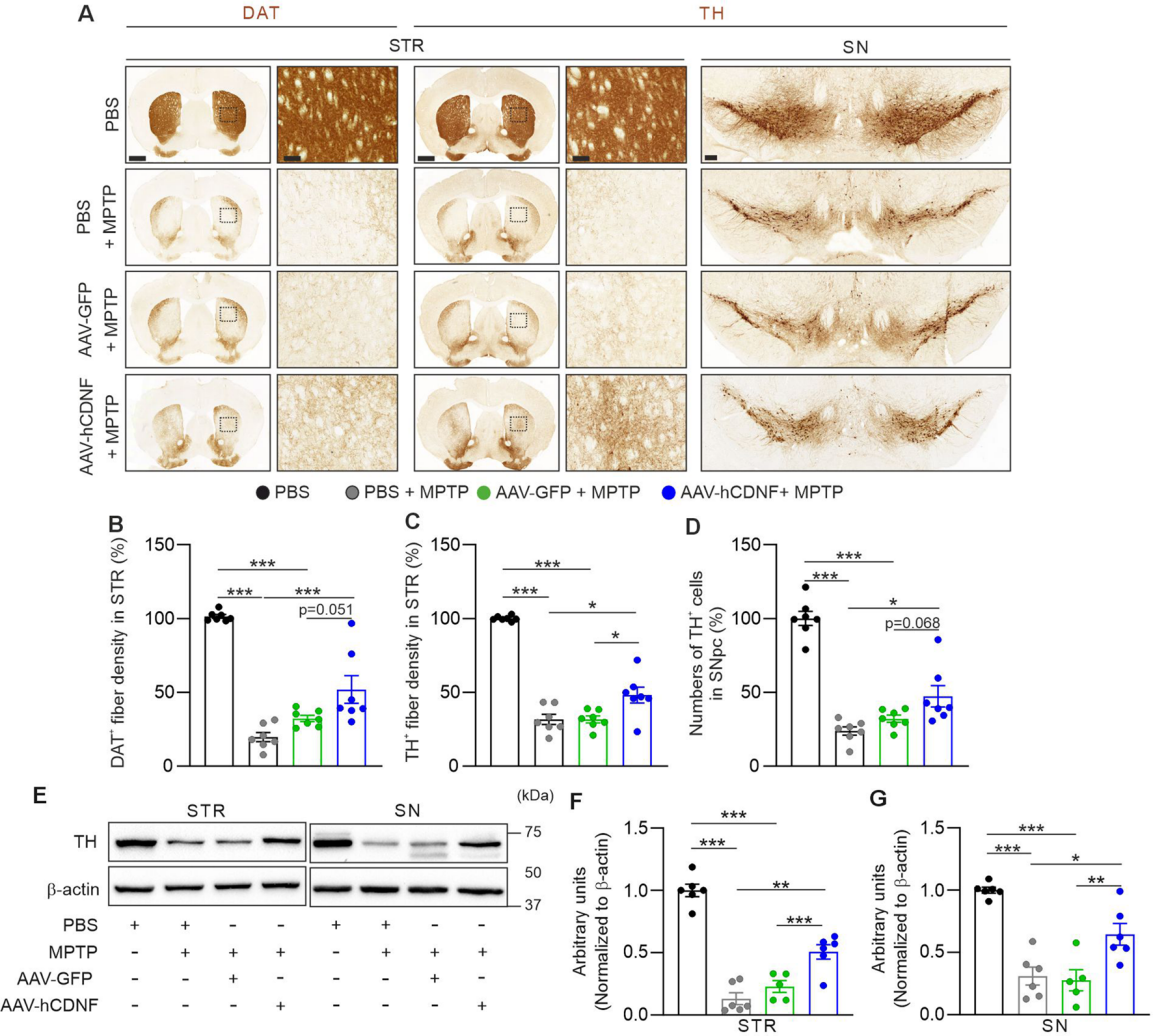
hCDNF attenuates the degeneration of the nigrostriatal dopamine pathway in the acute MPTP model. (**A**) Immunohistochemical staining of STR sections for DAT (left) and TH (centre) and of SN sections for TH (right). Enlarged images of the dotted rectangle in the STR. Scale bars: 1 mm for the STR, 100 μm for the enlarged image of the STR, and 200 μm for the SN. (**B**) Quantification of the density of DAT+ fibres in the STR. (**C**) Quantification of the density of TH+ fibres in the STR. (**D**) Quantification of the number of TH+ neurons in the SNpc. (**E**) Immunoblot image of TH and β-actin in the STR and SN. β-Actin was used as an internal control. (**F**) Quantification of TH expression in the STR normalized to β-actin expression. (**G**) Quantification of TH expression in the SN normalized to β-actin expression. (**B**–**D**) *n* = 7 for the PBS group, *n* = 7 for the PBS + MPTP group, *n* = 7 for the AAV-GFP + MPTP group, and *n* = 7 for the AAV-hCDNF + MPTP group. (**F**,**G**) *n* = 6 for the PBS group, *n* = 6 for the MPTP group, *n* = 5 for the AAV-GFP + MPTP group, and *n* = 6 for the AAV-hCDNF + MPTP group. One-way ANOVA followed by Tukey’s multiple comparisons post hoc test. All the data are expressed as the mean ± S.E.M. *p < 0.05, **p < 0.01, and ***p < 0.001.

MPTP treatment causes the loss of dopaminergic fibres and neurons in the nigrostriatal pathway at 7 days after administration^18,37^. Next, we evaluated the neuroprotective effect of hCDNF on the nigrostriatal dopaminergic pathway in an MPTP-induced PD model by immunohistochemical staining for the dopaminergic markers TH and DAT (Fig. 3A–D). The DAT+ (for PBS + MPTP: 80.3% ± 8.21%, AAV-GFP + MPTP: 67.7% ± 5.55%) and TH+ (for PBS + MPTP: 68.4% ± 9.02%, AAV-GFP + MPTP: 68.3% ± 6.2%) fibre densities in the striatum and the percentage of TH+ cells (for PBS + MPTP: 76.1% ± 7.74%, AAV-GFP + MPTP: 67.9% ± 6.41%) in the SN were reduced in the PBS + MPTP and AAV-GFP + MPTP groups compared with the PBS-treated group. The densities of DAT+ (48.1% ± 24.6%) and TH+ (51.8% ± 14.2%) fibres in the striatum and the number of TH+ cells (52.6% ± 19.2%) in the SN were significantly greater in AAV-hCDNF-treated mice, than in PBS + MPTP- and AAV-GFP + MPTP-treated mice (Fig. 3B–D). We verified the immunohistochemical data by Western blot analysis of the levels of TH (Fig. 3E–G). The levels of TH in the STR revealed by Western blot analysis were consistent with those revealed by immunohistochemistry (Fig. 3F), as were the TH levels in the SN shown by Western blot analysis (Fig. 3G). Taken together, the results of immunohistochemistry and Western blot analysis verify that hCDNF protected TH and DAT levelsin the nigrostriatal pathway in MPTP-treated mice.

### hCDNF reduces microglial IL-1β levels and alters microglial morphology in an MPTP mouse model

**Figure 4:**
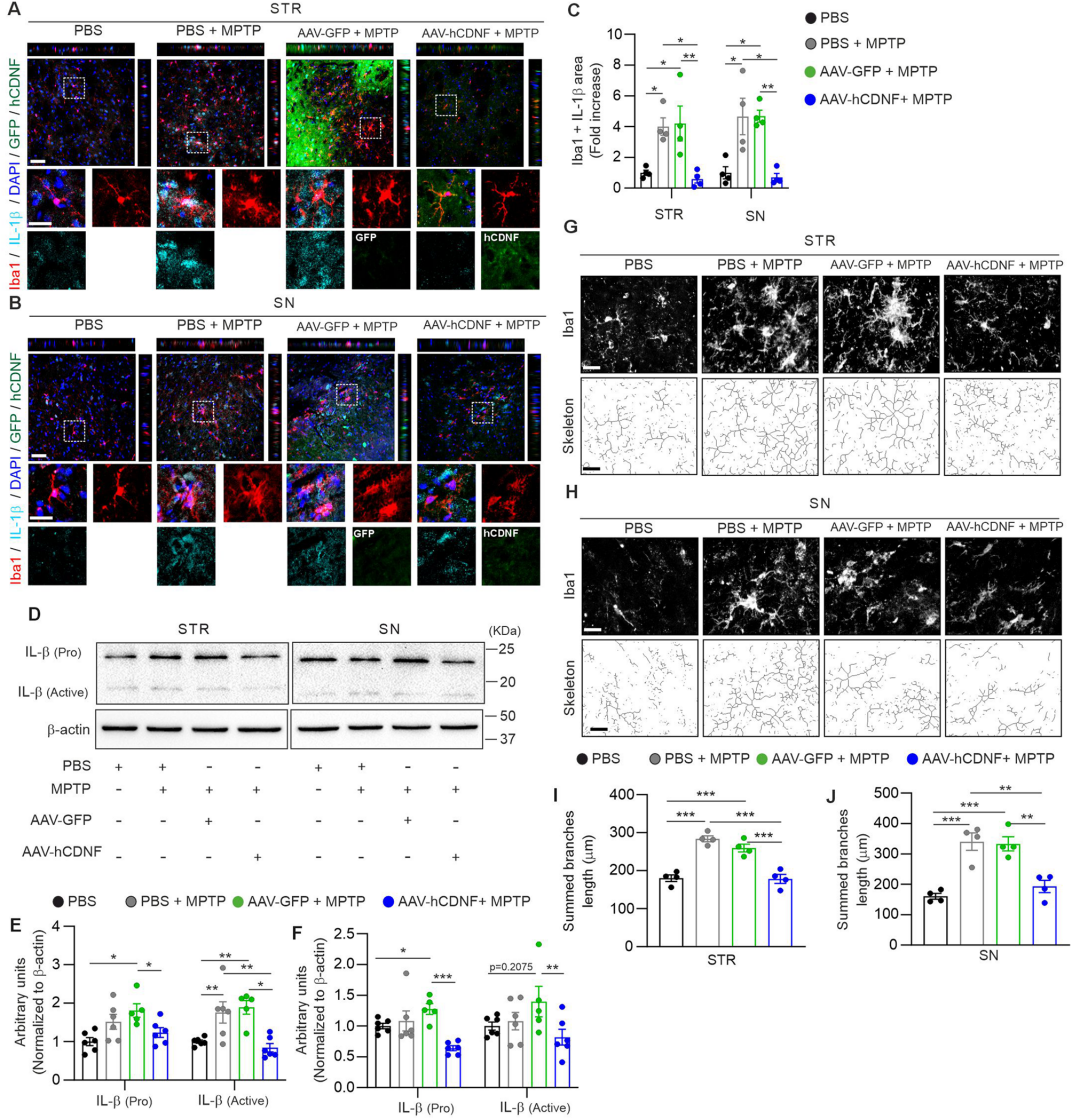
hCDNF decreases microglial IL-1β expression and normalizes Iba1-positive microglial morphology in the acute MPTP model. (**A**,**B**) Z-stack images of immunofluorescence double staining for Iba1 (red), IL-1β (cyan), DAPI (blue) and GFP or hCDNF (green) in the STR (upper panel, **A**) and SN (lower panel, **B**). The dotted white rectangles indicate the enlarged areas. Scale bar: 50 µm for the z-stack images and 25 µm for the enlarged images. (**C**) Quantification of the Iba1 + IL-1β pixel area in the STR and SN. (**D**) Western blot analysis of IL-1β and β-actin expression in the STR and SN. (**F**) Quantification of IL-1β expression in the STR normalized to β-actin expression. (**G**) Quantification of IL-1β expression in the SN normalized to β-actin expression. (**G**,**H**) Representative images for the analysis of microglial morphology with Iba1 staining (upper panels, grey) and skeletonized images (lower panels) of the STR (**G**) and SN (**H**). Scale bar, 20 µm. (**I**,**J**) Quantification of the Iba1-positive microglial branch length in the STR and SN. (**C**) STR: *n* = 16 images/4 mice for the PBS group, *n* = 16 images/4 mice for the PBS + MPTP group, *n* = 16 images/4 mice for the AAV-GFP + MPTP group, and *n* = 17 images/4 mice for the AAV-hCDNF + MPTP group; SN: *n* = 16 images/4 mice for the PBS group, *n* = 17 images/4 mice for the PBS + MPTP group, *n* = 16 images/4 mice for the AAV-GFP + MPTP group, and *n* = 17 images/4 mice for the AAV-hCDNF + MPTP group. (**E**,**F**) n = 6 for the PBS group, n = 6 for the MPTP group, n = 5 for the AAV-GFP + MPTP group, and n = 6 for the AAV-hCDNF + MPTP group. (**I**) *n* = 174 cells/4 mice for the PBS group, *n* = 249 cells/4 mice for the PBS + MPTP group, *n* = 168 cells/4 mice for the AAV-GFP + MPTP group, and *n* = 247 cells/4 mice for the AAV-hCDNF + MPTP group. (**J**) *n* = 178 cells/4 mice for the PBS group, *n* = 321 cells/4 mice for the PBS + MPTP group, *n* = 182 cells/4 mice for the AAV-GFP + MPTP group, and *n* = 219 cells/4 mice for the AAV-hCDNF + MPTP group. (**C**,**F**,**G**) One-way ANOVA followed by Tukey’s multiple comparisons post hoc test. All the data are expressed as the mean ± S.E.M. *p < 0.05, **p < 0.01, and ***p < 0.001.

Neuroinflammation mediated by resident glial cells, such as microglia and astroglia, is considered a major pathophysiological feature of PD^19,42,43^. Activated microglia, astroglia, and increased levels of proinflammatory cytokines, such as IL-1β, have been reported in the MPTP mouse model of PD and in the brains of PD patients^44,45^. We investigated whether AAV-hCDNF attenuates neuroinflammation in MPTP-treated mice. To study the effect of hCDNF on neuroinflammation in MPTP-treated mice, we examined whether hCDNF could reduce in neuroinflammation in MPTP-treated mice by double immunofluorescence staining of brain sections adjacent to those shown in Fig. 2. First, we focused on the colocalization of Iba1 and IL-1β in the STR and SNpc. The colocalization of IL-1β and Iba1 was significantly greater in the PBS + MPTP and AAV-GFP + MPTP groups than in the PBS group. The area of colocalized IL-1β + Iba1 was significantly decreased in AAV-hCDNF-treated mice compared with MPTP- and AAV-GFP + MPTP-treated mice (Fig. 4A–C). Additionally, the immunofluorescence data were verified by Western blot analysis of the level of IL-1β. IL-1β exists in the inactive form called pro-IL-1β, which is subsequently transformed into the mature and active form (active IL-1β) by caspase-1^46^. In both the STR and SN, the levels of pro- and active IL-1β were increased in AAV-GFP + MPTP-treated mice. In AAV-hCDNF-injected mice, the levels of pro-IL-1β and active IL-1β were significantly decreased (Fig. 4D–F). Western blot analysis of IL-1β levels verified the results obtained by immunohistochemistry. Moreover, to analyse microglial morphology, we quantified the branch length of Iba1-positive cells (Fig. 4G–J). The branch length in the STR and SN was greater in the PBS + MPTP- and AAV-GFP + MPTP-treated groups than in the PBS-treated group, while in the AAV-hCDNF-treated mice, the length of the branches was significantly reduced (Fig. 4I,J). These results suggest that AAV-hCDNF reduced the number of cells coexpressing IL-1β and Iba1 and reduced microglial branching in MPTP-treated mice.

### hCDNF reduces astroglia-derived IL-1β and the number of A1 astroglia in the MPTP mouse model

**Figure 5:**
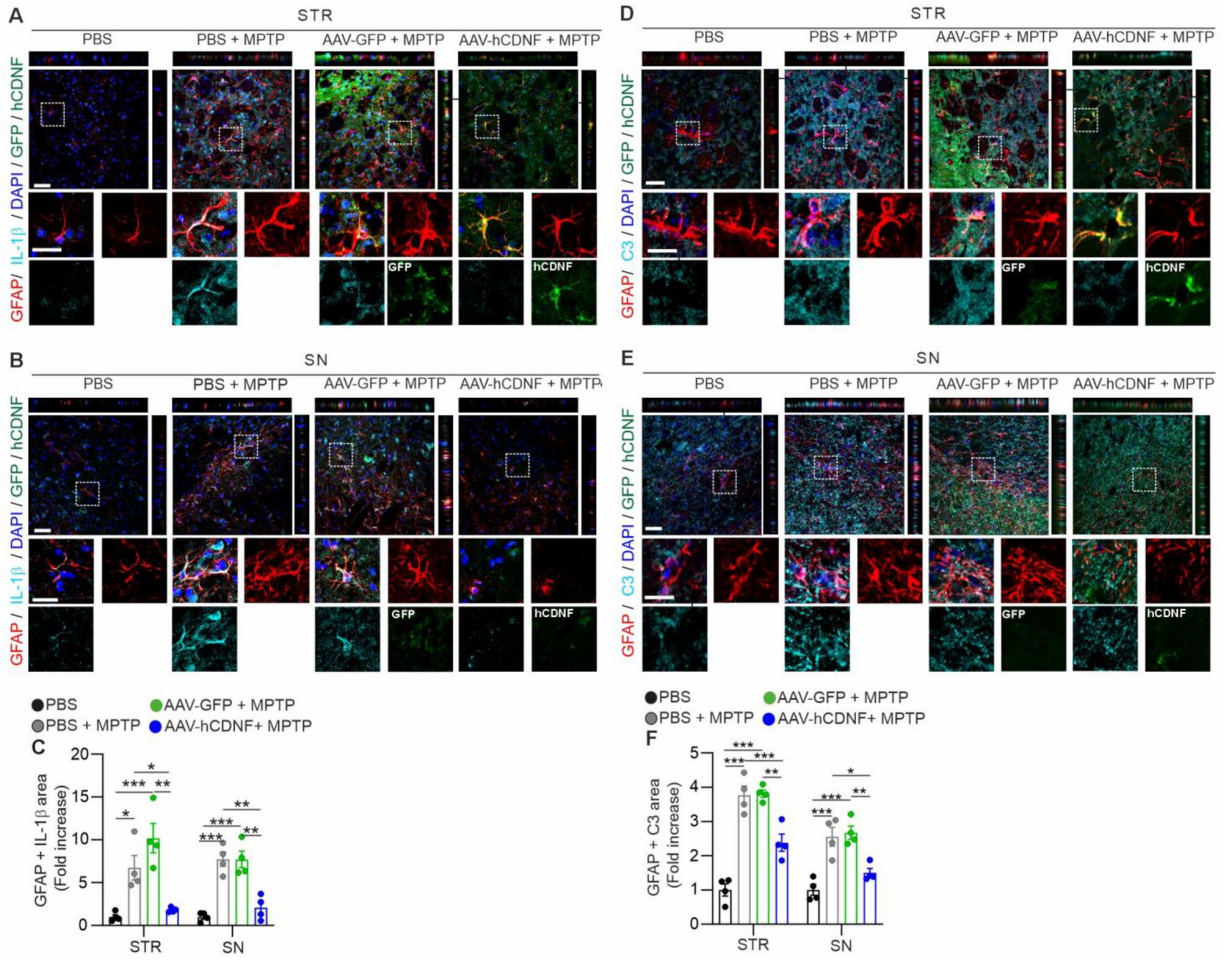
hCDNF decreases astroglial IL-1β and C3 expression in the acute MPTP model. (**A**,**B**) Z-stack images of immunofluorescence double staining for GFAP (red), IL-1β (cyan), DAPI (blue) and GFP or hCDNF (green) in the STR (upper panel, **A**) and SN (lower panel, **B**). The dotted white rectangles indicate the enlarged areas. Scale bar: 50 µm for the z-stack images and 20 µm for the enlarged images. (**C**) Quantification of the GFAP + IL-1β pixel area in the STR and SN. (**D**,**E**) Z-stack images of immunofluorescence double staining for GFAP (red), C3 (cyan), DAPI (blue) and GFP or hCDNF (green) in the STR (upper panel, **C**) and SN (lower panel, **D**). The dotted white rectangles indicate the enlarged areas. Scale bar: 50 µm for the z-stack images and 25 µm for the enlarged images. (**F**) Quantification of the GFAP + C3 pixel area in the STR and SN. (**C**) STR: *n* = 16 images/4 mice for the PBS group, *n* = 14 images/4 mice for the PBS + MPTP group, *n* = 15 images/4 mice for the AAV-GFP + MPTP group, and *n* = 16 images/4 mice for the AAV-hCDNF + MPTP group; SN: *n* = 16 images/4 mice for the PBS group, *n* = 16 images/4 mice for the PBS + MPTP group, *n* = 16 images/4 mice for the AAV-GFP + MPTP group, and *n* = 16 images/4 mice for the AAV-hCDNF + MPTP group. (**F**) STR: *n* = 15 images/4 mice for the PBS group, *n* = 13 images/4 mice for the PBS + MPTP group, *n* = 12 images/4 mice for the AAV-GFP + MPTP group, and *n* = 13 images/4 mice for the AAV-hCDNF + MPTP group; SN: *n* = 15 images/4 mice for the PBS group, *n* = 13 images/4 mice for the PBS + MPTP group, *n* = 14 images/4 mice for the AAV-GFP + MPTP group, and *n* = 12 images/4 mice for the AAV-hCDNF + MPTP group. (**C**,**F**) One-way ANOVA followed by Tukey’s multiple comparisons post hoc test . All the data are expressed as the mean ± S.E.M. *p < 0.05, **p < 0.01, and ***p < 0.001.

Astroglia also play an important role in neuroinflammation associated with PD^19,44^. First, we tested whether AAV-hCDNF reduces astroglia-related neuroinflammation in the MPTP mouse model of PD by double immunofluorescence. The colocalization area of GFAP and IL-1β immunolabelling was significantly greater in PBS + MPTP-treated and AAV-GFP + MPTP-treated mice than in PBS-treated mice (Fig. 5A–C). Compared with the PBS + MPTP and AAV-GFP + MPTP groups, the colocalization of GFAP and IL-1β was decreased in the AAV-hCDNF group (Fig. 5C). In addition, we examined whether A1 astroglia in the MPTP mouse model are regulated by hCDNF. To analyse the A1 polarization of astroglia, brain sections were stained for GFAP and C3, which are A1 astroglial markers. The colocalization area of GFAP and C3 was significantly greater in PBS + MPTP- and AAV-GFP + MPTP-treated mice than in PBS-treated mice (Fig. 5D–F). Compared with that in PBS + MPTP- and AAV-GFP + MPTP-treated mice, the colocalization of C3 + GFAP in AAV-hCDNF-treated mice significantly decreased (Fig. 5F). These results indicate that hCDNF reduces astroglial IL-1β and C3 expression in the MPTP mouse model of PD.

### hCDNF reduces CHOP and GRP78 expression in astroglia in the MPTP mouse model of PD

**Figure 6:**
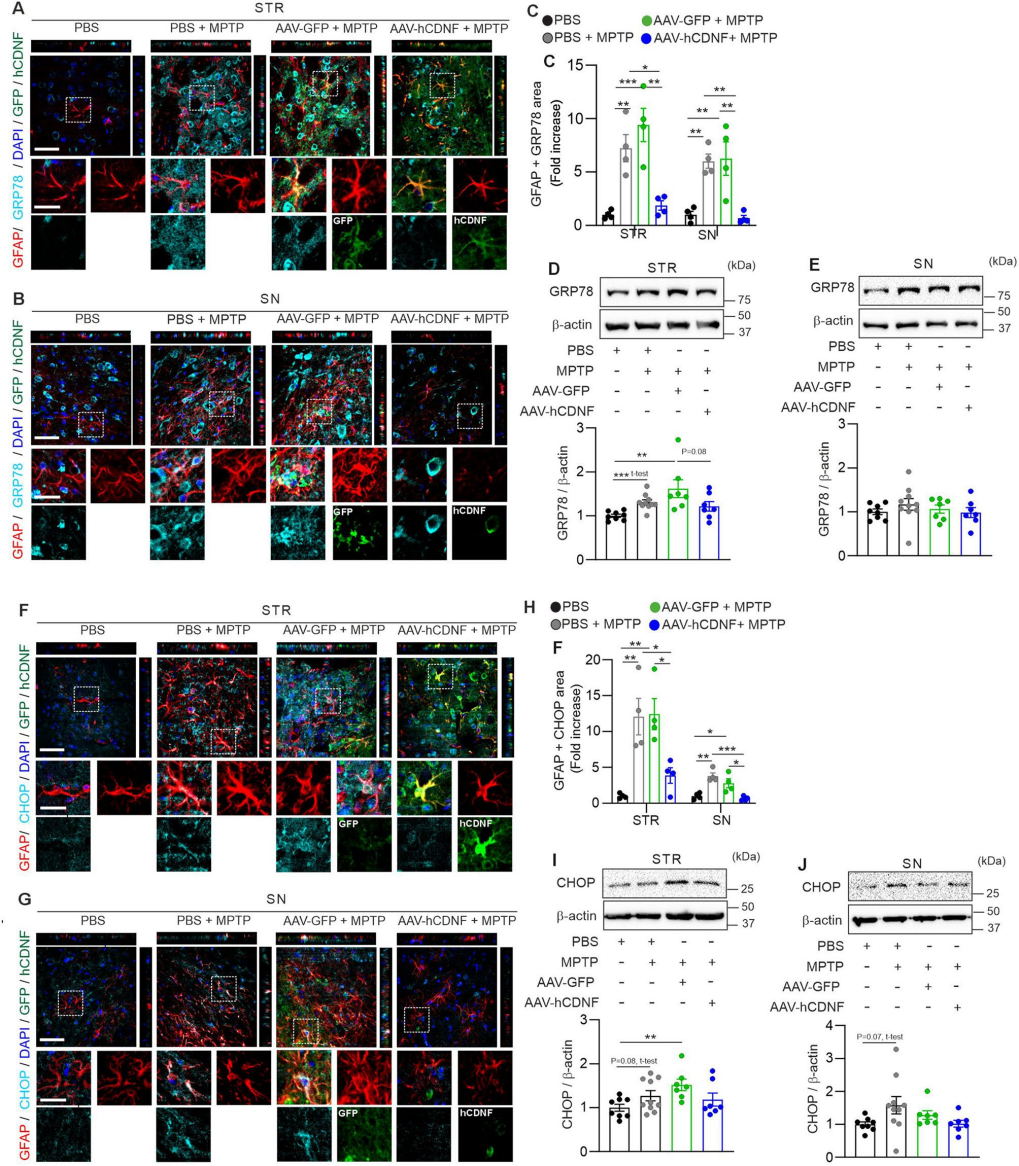
hCDNF inhibits ER stress markers in astroglia in the acute MPTP model. (**A**,**B**) Z-stack images of immunofluorescence double staining for GFAP (red), GRP78 (cyan), DAPI (blue) and GFP or hCDNF (green) in the STR (upper panel, **A**) and SN (lower panel, **B**). The dotted white rectangles indicate the enlarged areas. Scale bar: 50 µm for the z-stack images and 25 µm for the enlarged images. (**C**) Quantification of the GFAP + GRP78 pixel area in the STR and SN. (**D**,**E**) Western blot analysis of GRP78 and β-actin expression in the STR and SN. (**D**) Quantification of GRP78 expression in the STR and normalization to β-actin expression. (**E**) Quantification of GRP78 expression in the SN normalized to β-actin expression. (**F**,**G**) Z-stack images of immunofluorescence double staining for GFAP (red), CHOP (cyan), DAPI (blue) and GFP or hCDNF (green) in the STR (upper panel, **F**) and SN (lower panel, **G**). The dotted white rectangles indicate the enlarged areas. Scale bar: 50 µm for the z-stack images and 25 µm for the enlarged images. (**H**) Quantification of the GFAP + CHOP pixel area in the STR and SN. (**I**,**J**) Western blot analysis of CHOP and β-actin expression in the STR and SN. (**I**) Quantification of CHOP expression in the STR normalized to β-actin expression. (**J**) Quantification of CHOP expression in the SN normalized to β-actin expression. (**C**) STR: *n* = 15 images/4 mice for the PBS group, *n* = 13 images/4 mice for the PBS + MPTP group, *n* = 15 images/4 mice for the AAV-GFP + MPTP group, and *n* = 19 images/4 mice for the AAV-hCDNF + MPTP group; SN: *n* = 12 images/4 mice for the PBS group, *n* = 12 images/4 mice for the PBS + MPTP group, *n* = 14 images/4 mice for the AAV-GFP + MPTP group, and *n* = 14 images/4 mice for the AAV-hCDNF + MPTP group. (**H**) STR: *n* = 13 images/4 mice for the PBS group, *n* = 14 images/4 mice for the PBS + MPTP group, *n* = 12 images/4 mice for the AAV-GFP + MPTP group, and *n* = 19 images/4 mice for the AAV-hCDNF + MPTP group; SN: *n* = 13 images/4 mice for the PBS group, *n* = 12 images/4 mice for the PBS + MPTP group, *n* = 12 images/4 mice for the AAV-GFP + MPTP group, and *n* = 12 images/4 mice for the AAV-hCDNF + MPTP group. (**D**,**E**,**I**,**J**) n = 6 for the PBS group, n = 6 for the MPTP group, n = 5 for the AAV-GFP + MPTP group, and n = 6 for the AAV-hCDNF + MPTP group. (**C**–**E**,**G**,**I**,**J**) One-way ANOVA followed by Tukey’s multiple comparisons post hoc test. All the data are expressed as the mean ± S.E.M. *p < 0.05, **p < 0.01, and ***p < 0.001.

CDNF has a KTEL sequence in the C-terminus, which is known to be a signal for ER retention, and recently, CDNF was identified as a UPR pathway regulator in skeletal muscle in vivo^7,47^. Moreover, in genetic rodent models of ALS, hCDNF regulates the UPR pathway in motor neurons of the spinal cord^10^. Additionally, the upregulation of UPR pathway-associated proteins has been reported in the brains of PD model rodents^31^. Previous work has suggested that UPR signalling molecules, including GRP78 and CHOP, are activated in activated astroglia in the striatum of 3-nitropropionic acid (3-NP)-injected rats and in the spinal cord of contusion SCI model rats^48,49^. In addition, GRP78 and CHOP levels are increased in an in vitro   model of ischaemia in primary astroglia^50^. Therefore, we examined the effects of AAV-hCDNF on UPR pathway signalling in MPTP-induced PD mouse model. We investigated whether the astroglial levels of the UPR markers GRP78 and CHOP are altered by hCDNF in the MPTP mouse model of PD. The area of overlap between GRP78 and GFAP immunoreactivity was significantly greater in the PBS + MPTP- and AAV-GFP + MPTP-treated mice than in the PBS-treated mice (Fig. 6C). Colocalization of GRP78 and GFAP immunoreactivity in AAV-hCDNF-treated mice was significantly decreased compared with that in PBS + MPTP- and AAV-GFP + MPTP-treated mice (Fig. 6A–C). We verified the immunofluorescence results by Western blotting. GRP78 levels were increased in AAV-GFP + MPTP-treated mice. AAV-hCDNF treatment slightly reduced the level of GRP78 in the STR but not in the SN (Fig. 6D,E). Furthermore, we tested immunoreactivity for CHOP, whose expression is induced by ER stress and which is known to be a transcription factor in astroglia^49,50^. Compared with that in PBS-treated mice, the area of colocalization of CHOP and GFAP in PBS + MPTP- and AAV-GFP + MPTP-treated mice was significantly greater (Fig. 6H). CHOP immunoreactivity in astroglia was decreased in the AAV-hCDNF-treated group (Fig. 6F–H). Additionally, we tested the level of CHOP by Western blotting. CHOP levels in both the STR and SN tended to be increased in the MPTP and AAV-GFP + MPTP groups but slightly decreased in the AAV-hCDNF group, but the difference was not significant (Fig. 6I,J). These results showed that AAV-hCDNF alters the association of two ER stress markers, GRP78 and CHOP, within astroglia in the STR and SN in the MPTP-induced acute PD mouse model.

## Discussion

CDNF is known for its neurotrophic activity and protective and restorative effects. The effect of CDNF on neurological diseases, especially PD, has been widely studied^1,3,16–18,51^. The main result of this study is the finding that AAV-mediated delivery of hCDNF exerts beneficial effects in the MPTP mouse model of PD by improving motor function and preventing dopamine neuron loss in the nigrostriatal pathway. Additionally, we showed that AAV-hCDNF reduces the levels of proinflammatory cytokines (IL-1β and C3) and ER stress markers (GRP78 and CHOP) in glial cells.

The use of AAV vectors is efficient and safe method of gene delivery for gene-based therapies for CNS diseases, including PD^52^. In our study, we used AAV serotype 1 to deliver hCDNF into the mouse brain. Analysis of the efficiency of AAV-hCDNF and AAV-GFP transduction showed that hCDNF and GFP were expressed in the STR and SN of the brain at 2 and 6 weeks after AAV injection. Additionally, we found that TH-positive dopaminergic neurons in the SNpc were transduced with hCDNF. This could be explained by our previous results showing that exogenous hCDNF is retrogradely transported from the STR to the SN in vivo^2^. Moreover, AAV-mediated transgene delivery to the STR and SN did not significantly affect the density of TH+ and DAT+ fibres in the STR or the number of TH+ cells in the SN, indicating no overt toxicity. CDNF is known as an ER-resident protein because the C-terminus of CDNF has an ER retention signal sequence, KTEL, which allows it to reside in the lumen of the ER^36^. Considering the hCDNF sequence, we created the vector AAV-GFP, which has a signal peptide and a THPKTEL sequence that is the same as the last seven amino acids of CDNF, which is important for targeting the protein to and retaining it in the ER lumen. We confirmed that hCDNF and GFP colocalized with GRP78, a resident protein of the ER lumen. Additionally, AAV-GFP was found to be a suitable control for AAV-hCDNF in our study given that the protein is processed in a similar way to hCDNF, in contrast to cytosolic GFP (Fig. 1 and Supplementary Fig. 1).

The potential effectiveness of AAV2-hCDNF and AAV8-hCDNF for gene therapy has been tested in the 6-OHDA-induced PD rat model, and the results showed that hCDNF delivered with AAV was able to attenuate amphetamine-induced rotation behaviours and prevent the loss of dopaminergic fibres and neurons^14–17^. In this study, we first tested the effectiveness of gene therapy with AAV-hCDNF in the acute MPTP-induced PD model. This animal model exhibits specific degeneration of dopaminergic fibres and neurons in the nigrostriatal pathway due to MPTP toxicity, resulting in motor dysfunction similar to that observed in PD. In line with previous results, here, we showed that AAV-hCDNF mitigated the impairment of motor function in MPTP-treated mice, as evaluated by the rotarod and coat hanger tests (Fig. 2). Additionally, we first investigated whether AAV-hCDNF regulates gait dynamics in the acute MPTP mouse model. Imbalance during walking and impaired gait are commonly observed in PD patients and MPTP treated mice^41,53^. We analysed diverse gait parameters (Supplementary Table 1) and found that hCDNF had an effect on stride frequency. According to the literature, gait dynamics in the MPTP mouse model can be influenced by several factors, such as the type of MPTP model, the speed of the treadmill, and the observation time point. We used DigiGait to assess the gait of acute MPTP model mice at a speed of 20 cm/s at 6 days post-MPTP injection, which might be an unsuitable time point for testing overall gait dynamics parameters in this model (Fig. 2, Supplementary Fig. 3). Furthermore, in this animal model, injection of MPTP caused selective degeneration of dopaminergic neurons in the nigrostriatal pathway^18^. We explored the effect of AAV-hCDNF on the MPTP-induced damage to the nigrostriatal dopamine pathway. AAV-hCDNF protected the nigrostriatal dopamine pathway at 7 days post-MPTP injection. We verified the neuroprotective effects of AAV-hCDNF by performing immunoblotting for TH and DAT (Fig. 3). Our results showed that the protective effect of AAV-hCDNF in the acute MPTP-induced PD model was greater in the STR than in the SN (Fig. 2B–D). This could be explained by the efficiency of AAV-hCDNF transduction. While hCDNF was widely expressed in the STR, its expression in the SN was relatively low.

Neuroinflammation mediated by glial cells and infiltrating immune cells is considered the critical pathological feature of neurodegenerative diseases, including PD^43^. Glial activation plays a significant role in the onset of nigrostriatal degeneration in patients with PD and in mice treated with MPTP. When glial cells become active, they produce proinflammatory cytokines and chemokines, which leads to neuronal death. Our study focused on neuroinflammation, which is involved in PD pathogenesis, as a possible target of hCDNF in MPTP-treated PD model mice. IL-1β is a proinflammatory cytokine that regulates neuroinflammation and neurophysiological activities in the CNS^54^. In PD, the degeneration of dopamine fibres and neurons via the neuroinflammatory cascade has been linked to IL-1β derived from microglia, astroglia, and infiltrating immune cells^27,28,55^. In many in vivo studies, including ours, we detected an increase in IL-1β protein expression in the acute MPTP-induced PD model and found that IL-1β was expressed in activated microglia and astroglia in the STR and SN of MPTP-treated mice. Treatment with AAV-hCDNF significantly reduced the expression of IL-1β in microglia and astroglia in the STR and SN of MPTP-treated mice (Figs. 4, 5). Additionally, we detected IL-1β expression on CD68-positive microglia and CD45-positive infiltrating immune cells in MPTP-treated mice, and this expression of IL-1β was reduced in AAV-hCDNF + MPTP-treated mice (Supplementary Fig. 4). Moreover, we analysed the morphological phenotype of microglia and showed that the increase in the branch length of microglia in MPTP-treated mice was reversed by hCDNF treatment.

In many studies, astroglia has also shown to play important roles in brain functions such as neurotransmitter homeostasis, maintaining the structure of the BBB, and regulating neuroinflammation. According to a previous report^20^, after release from activated microglia, cytokines such as IL-1α, TNFα and C1q convert astroglia to the A1 and A2 phenotypes. A1 astroglia are considered potentially harmful. Component 3 (C3) is used as an A1 astroglial marker^19,20^. A1/A2 astroglial polarization has been observed in the postmortem brains of individuals with ALS, AD, MS, HD, and PD^20^. Moreover, the regulation of A1 astroglial polarization has been identified as a therapeutic target for PD^19^. We detected C3 in the astroglia of MPTP-treated mice. The increase in C3 and GFAP colocalization in MPTP-treated mice was prevented by hCDNF (Fig. 5). On the basis of this combined evidence, we tentatively speculate that hCDNF delivered with AAV exerts a neuroprotective effect by regulating microglia-/astroglia-mediated neuroinflammation.

CDNF regulates ER stress. According to recent studies, in the skeletal muscle of CDNF knockout mice, the expression of UPR-related proteins such as GRP78 and Xbp1s is upregulated^7^, and in HEK293-T cells, CDNF modulates thapsigargin-induced ER stress^56^. Additionally, in diseases, such as ALS, CDNF rescues motor neurons in the spinal cord through the modulation of UPR signalling^10^. In a study of PD, the protein and mRNA levels of GRP78 and CHOP in PD patients and PD patients with dementia were found to be altered in the brain, specifically the temporal cortex, cingulate gyrus and SN, but not in the cerebrospinal fluid (CSF)^32,33^. Additionally, CDNF-overexpressing astroglia alleviate tunicamycin-induced cytotoxicity^57^. Currently, no results have been reported regarding the effect of CDNF on the astrocytic UPR pathway in the MPTP mouse model of PD. We found that the GRP78 and CHOP levels in astroglia were increased by MPTP and attenuated by hCDNF (Fig. 6). We also measured the levels of GRP78 and CHOP by immunoblotting. However, immunoblotting revealed that GRP78 and CHOP levels did not significantly differ between the groups. A recent finding might explain this: In the acute MPTP-induced PD model, glial activation and proinflammatory cytokine levels peak at 1 and 3 days after MPTP treatment^58^. In this study, we focused on the mechanism underlying the effect of hCDNF 7 days after MPTP treatment. This time point is suitable for investigating neuroprotective effects and motor function impairment, but it has limitations for the examination of microglial and astrocyte functions.

Consistent with previous results, we showed a neuroprotective effect of exogenous hCDNF delivered using an AAV construct in an MPTP mouse model of PD. Furthermore, we found that AAV-hCDNF improved motor coordination and prevented the degeneration of dopaminergic fibres and neurons in the nigrostriatal pathway by attenuating glial cell-derived neuroinflammation and ER stress. In other words, the modulation of glial cell-mediated neuroinflammation and UPR pathway signalling may be one of the mechanisms underlying the neurotrophic effect of CDNF in the MPTP mouse model PD. Our results demonstrate the beneficial effect for hCDNF on behaviour and neural function in a mouse model of PD when delivered by an AAV vector and provide new evidence for the anti-inflammatory and ER stress-regulating effects of CDNF in the MPTP mouse model of PD.

## Materials and methods

### Study design

All animal experiments were approved by the Finnish National Board of Animal Experiments and were carried out according to the European Community guidelines for the use of experimental animals with the license number (ESAVI/12830/2020). Male C67BL/6 mice (9–10 weeks old and weighing approximately 28 g) were utilized in these experiments since sex differences in MPTP neurotoxicity have been reported^59^. The mice were obtained from Envigo (Netherlands) and housed in groups of three to four on a 12:12 light:dark cycle at an ambient temperature of 22 °C, and food and water were available ad libitum. The mice were given ID numbers and randomly divided into the following experimental groups according to body weight or behaviour on the rotarod test: for characterization of AAV toxicity in naïve mice, the PBS (*n* = 10, 2 mice were used for Western blot analysis, 8 mice were used for IHC analysis), AAV-GFP (*n* = 10, 3 mice were used for Western blot analysis, 8 mice were used for IHC analysis), and AAV-hCDNF (*n* = 13, 3 mice were used for Western blot analysis, 10 mice were used for IHC analysis) groups; for analysis of the effect of AAV-hCDNF in the MPTP mouse model, the PBS (*n* = 13, 6 mice were used for Western blot analysis, 7 mice were used for IHC analysis), MPTP (*n* = 13, 6 mice were used for Western blot analysis, 7 mice were used for IHC analysis), AAV-GFP + MPTP (*n* = 12, 5 mice were used for Western blot analysis, 7 mice were used for IHC analysis), and AAV-hCDNF + MPTP (*n* = 13, 6 mice were used for Western blot analysis, 7 mice were used for IHC analysis) groups. The animals were divided between 2 independent replicate experiments as follows: in the PBS group (n = 13),7 mice were used for the 1st experiment, and 6 mice used for the 2nd experiment;, in the PBS + MPTP group (n = 13),6 mice were used for the 1st experiment, and 7 mice were used for the 2nd experiment; in the AAV-GFP + MPTP group (n = 12),8 mice were used for the 1st experiment, and 4 mice were used for the 2nd experiment; and in the AAV-hCDNF + MPTP group (n = 13),8 mice were used for the 1st experiment, and 5 mice were used for the 2nd experiment. Stereotaxic surgeries were carried out under isoflurane anaesthesia (Vetflurane® 1000 mg/g, Virbac, France; 4% for induction, 2–4% for maintenance). The mice were placed in a stereotaxic frame (Stoelting) and were injected with 0.5 µl of AAV-GFP (Conc 1.93 × 10^12^ vg/ml), AAV-hCDNF (Conc 1.71 × 10^12^ vg/ml), or phosphate-buffered saline (PBS, B. Braun Medical, Finland) as a control into the bilateral striata (anterior–posterior (AP): + 0.5 mm; medial–lateral (ML): + / − 1.8 mm; dorsal–ventral (DV): − 3.5 mm relative to the bregma), according to “The Mouse Brain in Stereotaxic Coordinates”^60^. Injections were performed using a 10 µl NanoFil syringe (33G, World Precision Instruments, USA), and the infusion rate for virus injection was 0.1 µl/min. The needle was kept in the brain for 5 min to allow diffusion and minimize the backflow of the viral vectors. The animals received 5 mg/ml Carprofen (Ramydal^®^, Zoetis) for postoperative pain relief. To characterize AAV toxicity, the animals were sacrificed 2, 4 or 6 weeks after AAV injection (Fig. 1, Supplementary Fig. 1). To investigate the effect of AAV-hCDNF on the MPTP mouse model, four weeks after AAV-GFP, AAV-hCDNF, or PBS treatment, the mice were injected with MPTP. MPTP (20 mg/kg free-base; M0896, Sigma) was dissolved in PBS (B. Braun Medical, Finland) and injected into mice intraperitoneally (i.p.) four times at 2-h intervals during the light cycle^18,37^. After the last injection of MPTP, the mice were given soft food for 3 days, and the animals were housed in groups of three. After the mice were given MPTP injections, their behaviour was tested after 5 and 6 days. On day 5, the rotarod and open field tests were performed, while on day 6, the coat-hanger test and DigiGait analysis were performed (Fig. 2A).

### Production of AAV vectors

pscAAV CMV-IE hCDNF (Addgene# 188538, RRID: Addgene_188538) was constructed by replacing iRFP in pscAAV CMV-IE iRFP, a self-complementary AAV backbone with a CMV promoter driving iRFP, with the coding region of hCDNF. The resulting vector is referred to as “AAV-hCDNF” throughout this manuscript. pscAAV CMV-IE-secreted EGFP-THPKTEL WPRE (Addgene# 188539, RRID: Addgene_188539) was constructed by flanking the coding region of EGFP with the signal peptide sequence of CDNF at the N-terminus and with the CDNF ER retention sequence (THPKTEL) of CDNF at the C-terminus. The resulting coding sequence of sigpep-eGFP-THPKTEL as well as the woodchuck hepatitis virus post-transcriptional regulatory element (WPRE) was inserted into the same AAV backbone as AAV-hCDNF as described above^61,62^. The resulting vector is referred to as “AAV-GFP” throughout this manuscript. All cloning steps involved ligation-independent cloning (In-Fusion, Takara). The plasmids were propagated in Stbl3 cells (Invitrogen) and verified by restriction digestion/fragment analysis and Sanger sequencing prior to viral vector packaging. AAV vectors were produced as previously described^63^. All vectors were produced using serotype 1 capsid proteins and titered by droplet digital PCR.

### Behavioural analysis

To investigate motor deficits, three behavioural tests were used. For the open field test (ENV-520, Med Associates Inc.), the mice were placed in an activity chamber (chamber size: 28 cm × 28 cm), spontaneous locomotor activity was recorded for 1 h, and the distance travelled and number of vertical movements were subsequently analysed. For the rotarod test (Ugo Basile, Italy), the mice were tested on an accelerating rotating rod (from 4 to 40 rpm for a maximum of 300 s), and the latency to fall off the rod was recorded^64^. The coat hanger test was performed as previously described with slight modifications^13,65,66^. Briefly, a triangular-shaped wire hanger consisting of two diagonal sidebars on the left and right and a horizontal wire was used. The hanger was suspended at a height of 30 cm. The mice were placed on the middle of the horizontal wire and allowed to grip the hanger with their forepaws. Three parameters were analysed: the coat hanger score, the time spent hanging on the coat hanger and the time to reach the edge of the diagonal bar. The cut-off time for this assessment was 60 s. The coat hanger score was assigned as follows: (0) the mouse fell off in less than 10 s, (1) the mouse stayed on for more than 10 s and hung on the coat hanger, (2) the mouse placed one hind limb on the coat hanger, (3) the mouse put two hind limbs on the coat hanger, (4) the mouse wrapped its tail around the coat hanger and put both hind limbs on it, and (5) the mouse climbed to the top of the coat hanger (the highest score was achieved, for example, if the mouse had one hind limb and tail on the coat hanger)**.** The behaviour tests were carried out once, and 2 independent replicates of all tests except for the coat hanger test were performed. All behaviour tests were carried out from 8 am to 4 pm (during the light cycle).

### Analysis of gait dynamics

The DigiGait apparatus (Ventral Plane Imaging (VPI) Technology, USA) was used to assess walking and gait dynamics by analysing the digitalized paw and gait data of rodents^9,67,68^. Before the test, the mice were pretested on the treadmill of the DigiGait apparatus to determine the optimal speed and incline at which the mice were able to run forwards without stopping, walking backwards, or jumping. Based on published reports and preliminary test results, a speed of 20 cm/s and an incline of zero degrees was used for the test. The mice were recorded as they walked on a transparent treadmill for more than 10 s. Gait and paw data were digitalized from the recorded video, and approximately 3 s of video clips were used to analyse a variety of gait parameters (Supplementary Table 1). Multiple mice were excluded from the DigiGait analysis because they were not able to walk forwards and frequently stopped on the treadmill (the number of animals excluded was 1 for the PBS group 1 for the MPTP group, and 1 for the AAV-hCDNF + MPTP group).

### Immunohistochemistry

The mice were sacrificed 7 days after MPTP administration via transcardial perfusion after anaesthesia with sodium pentobarbital (Euthanimal Vet, Alfasan, Netherlands). Afterwards, the brain was fixed with 4% paraformaldehyde (PFA, Sigma) for 24 h and cryoprotected with 20% sucrose (Fisher). After the brains were frozen, the striatum (STR) was cryosectioned at 40 µm thickness, and the substantia nigra (SN) was cryosectioned at 35 µm thickness; all sections were collected in six separate series. Briefly, for DAB staining, the tissues were incubated with 0.3% H_2_O_2_ in PBS for 15 min to inactivate endogenous peroxidases. After washing with PBS three times, the tissues were incubated with blocking solution (0.2% Triton X-100/1% bovine serum albumin in PBS) for 1 h. Thereafter, the tissues were incubated overnight at 4 °C with primary antibody. The primary antibodies used were rabbit anti-GFP (1:4000, Invitrogen, A-11122, RRID:AB_221569), mouse anti-human CDNF (hCDNF, 1:1000, Icosagen, 302–100, RRID:AB_11134475), rabbit anti-tyrosine hydroxylase (TH, 1:2000, Pel-Freez Biologicals, P40101, RRID:AB_2617184) and rat anti-dopamine transporter (DAT, 1:1000, Santa Cruz, sc-32258, RRID:AB_627400). The next day, the tissues were incubated with the corresponding biotinylated secondary antibodies (biotinylated anti-mouse (1:400, BA-9200, Vector Laboratories Inc., RRID:AB_2336171), biotinylated anti-rat (1:400, BA-9400, Vector Laboratories Inc., RRID:AB_2336202), and biotinylated anti-rabbit (1:400, BA-1000, Vector Laboratories Inc., RRID:AB_2313606)) for 1 h and then incubated with avidin–biotin complex solution (PK-6100, Vector Laboratories Inc., RRID:AB_2336819) for 1 h. The antibody-antigen signals were visualized using 3,30-diaminobenzidine (DAB, Vector Laboratories Inc., RRID:AB_2336382). For Nissl staining, GFP- and CDNF-stained tissues were mounted on glass microscope slides and incubated with Nissl solution (0.1% Nissl) for 2 h. The glass slides were scanned using a Pannoramic 250 slide scanner (3DHistech, Hungary, RRID:SCR_022184) at 20 × magnification and with multilayer focus (extended focused). Then, images were captured using CaseViewer 2.4 instrument (3DHistech, RRID:SCR_017654) for image analysis. For immunofluorescence staining, the tissues were blocked and permeabilized with buffer (0.2% Triton X-100 with 5% donkey serum in PBS) for 1 h and then incubated with primary antibodies overnight at 4 °C. The primary antibodies used were rabbit anti-GFP, mouse anti-hCDNF, rabbit anti-hCDNF (1:1000, Icosagen, 300–100, RRID:AB_11140157), mouse anti-C/EBP homologous protein (CHOP, 1:500, Thermo Fisher, MA1-250, RRID:AB_2292611), rabbit anti-TH, mouse anti-TH (1:1000, Millipore, MAB318, RRID:AB_2313764), rabbit anti-glucose regulated protein 78 (GRP78, 1:2000, abcam, ab21685, RRID:AB_2119834), goat anti-glial fibrillary acidic protein (GFAP, 1:500, Novus Biological, NB100-53809, RRID:AB_829022), goat anti-Ionized calcium-binding adaptor molecule 1 (Iba1, 1:500, Wako, 011-27991), rabbit anti-complement 3 (C3, 1:400, ABclonal, A13283, RRID:AB_2760135), rat anti-mouse CD68 (FA-11, 1:200, Biolegend, 137007, RRID:AB_10575299), rat anti-mouse CD45 (IBL-3/16, 1:200, Bio-Rad, MCA1388, RRID:AB_321729) and rabbit anti-Interleukin-1β (IL-1β, 1:500, Abcam, ab9722, RRID:AB_308765). The following day, the tissues were rinsed with PBS and then incubated with the following fluorescence-conjugated corresponding secondary antibodies: Alexa Fluor™ 488-conjugated donkey anti-rabbit (1:400, Thermo Fisher, A-21206, RRID:AB_2535792), Alexa Fluor™ 647-conjugated donkey anti-mouse (1:400, Thermo Fisher, A-31571, RRID: AB_162542), and Alexa Fluor™ 568-conjugated donkey anti-goat (1:400, Thermo Fisher, A-11057, RRID:AB_142581). After 1 h of incubation, the tissues were rinsed with PBS and incubated with 6-diamidino-2-phenylindole (DAPI, 1:1000, Thermo Fisher) for visualization of the nuclei and counterstaining.

### Immunoblotting

The animals were euthanized in CO2 gas, and brain samples containing the STR and SN were dissected on dry ice. First, 5 mg of fresh sample was homogenized and lysed in 300 µl of lysis buffer (150 mM NaCl, 1% NP-40, 0.5% sodium deoxycholate, 1% SDS, and 50 mM Tris–Cl, (pH 8.0)) containing protease and phosphatase inhibitors (Roche). The lysates were incubated for 1 h at 4 °C and then centrifuged at 13,500 rpm for 20 min at 4 °C, after which the supernatants were transferred to a fresh tube. Protein concentrations were determined using a DC protein assay kit (Bio-Rad). Next, the samples were mixed with 4× Laemmli buffer (Bio-Rad) and boiled at 95 °C for 5 min. Finally, 20 µg (STR) or 10 µg (SN) of the sample was separated by sodium dodecyl sulfate‒polyacrylamide gel electrophoresis (SDS‒PAGE) and transferred to polyvinylidene difluoride membranes with a 0.45 µm pore size (PVDF, Millipore). The membranes were blocked with 5% skim milk (Valio, Finland) or 5% BSA (Sigma) in TBS-T (0.1% Tween-20 in Tris-buffered saline) for 1 h at room temperature (RT) and incubated with primary antibodies at 4 °C overnight. The primary antibodies used were rabbit anti-GFP (1:2000), mouse anti-hCDNF (1:1000), rabbit anti-TH (1:2000), rabbit anti-IL-1β (1:500), mouse anti-CHOP (1:1000), rabbit anti-GRP78 (1:1000), and mouse anti-β-actin (β-actin, 1:5000, Santa Cruz, sc-47778, RRID:AB_626632). The next day, the membranes were rinsed with TBS-T and incubated with the corresponding horseradish peroxidase (HRP)-conjugated secondary antibodies (1:3000, HRP-conjugated goat anti-mouse immunoglobulins, P0447, RRID:AB_2617137 or HRP-conjugated goat anti-rabbit immunoglobulin, P0448, RRID:AB_2617138) for 1 h at RT. The protein bands were visualized with Pierce ECL buffer (Pierce, Thermo Scientific, USA), and images were acquired and analysed with the ChemiDoc™ MP system (Bio-Rad, RRID:SCR_019037). The membranes were stripped with stripping buffer (Thermo Fisher Scientific, #21059) and reblotted with other primary antibodies.

### Imaging and analysis

Photomicrographs of the tissues were scanned at a resolution of 0.22 µm/pixel using a Pannoramic 250 instrument (3DHistech, Hungary); images of TH-stained SN tissues were taken at 20 × with an extended focus, and images of TH-, DAT-, GFP- and hCDNF-stained STR tissues were taken at 20× with a single layer focus. To quantify the density of TH^+^ and DAT^+^ fibres in the striatum and the distribution of GFP and hCDNF in the striatum, Fiji ImageJ (Fiji ImageJ 1.53 ver., RRID:SCR_003070) was used to analyse the density of TH^+^ and DAT^+^ fibres in six to eight coronal sections between + 1.4 and − 0.2 mm from bregma according to the brain atlas^60^, and the areas of GFP and hCDNF were analysed using CaseViewer 2.4 (3DHistech, RRID:SCR_017654). Furthermore, the density of TH^+^ and DAT^+^ fibres in different areas of the caudate putamen (the dorsal-lateral, dorsal-medial, ventral-lateral, and ventral-medial areas) was analysed, and the staining intensity in the corpus callosum area was subtracted to control for variations in background illumination. The PBS-treated group was used as a control^13,18^. For counting TH cell bodies in the SN, digitalized images were uploaded to the Cloud-based Aiforia^®^ platform (Aiforia™, Fimmic Oy, Helsinki, Finland) and counted using an automated deep conventional neural network algorithm. TH^+^ cell bodies in six SN tissues from approximately − 2.8 mm, − 2.92 mm, − 3.08 mm, − 3.16 mm, − 3.28 mm, and − 3.4 mm from bregma according to the brain atlas^60^ were counted for each animal. The PBS-treated group was used as a control^69,70^. For immunofluorescence staining, fluorescence images were acquired with an LSM700 microscope (Carl Zeiss) through an LCI Plan-Apochromat 25x/0.8 Imm Korr DIC M27 objective or LCI Plan-Neofluar 63x/1.3 Imm Corr objective with 80% glycerol, and all images were obtained through Z-stack imaging (2 to 3 µm interval) of a random area of the STR and SNpc and processed with maximum intensity projection. The images that were processed with maximum intensity projection and the adjusted threshold were used for the analysis of the pixel area using Fiji ImageJ (Fiji ImageJ 1.53 ver., RRID:SCR_003070). The colocalization of Iba1 with IL-1β, GFAP with IL-1β, GFAP with C3, GFAP with GRP78, and GFAP with CHOP was evaluated using the colocalization plug-in in Fiji ImageJ. Z-stack images of Iba1 staining were converted to skeletonized images using Skeletonize Plug-in, and then skeletonized images were analysed using the skeleton 2D/3D plug-in (developed by and maintained here: https://imagej.net/plugins/analyze-skeleton/?amp=1)^71^. We determined the total branch length from the results and normalized it to the number of Iba1^+^ cells.

### Statistical analysis

All the statistical analyses were conducted with GraphPad Prism 9 software (GraphPad Software, United States, RRID:SCR_002798). Statistical significance was determined by one-way followed by Tukey’s post hoc test for multiple comparisons. All values are presented as the mean ± standard error of the mean (SEM). The significance level is indicated by the number of asterisks (*p < 0.05, **p < 0.01, and ***p < 0.001). All the statistical analyses are presented in Supplementary Table 2.

### Supplementary Information

Supplementary Information.

## Data Availability

The raw data supporting the conclusions of this article will be made available by the authors and are available from the corresponding author on reasonable request.
