# Supplementary material for: Delivery of CDNF by AAV-mediated gene transfer protects dopamine neurons and regulates ER stress and inflammation in an acute MPTP mouse model of Parkinson’s disease
Source: Sci Rep. 2024 Jul 17;14:16487. doi: 10.1038/s41598-024-65735-5 (PMC11254911; doi:10.1038/s41598-024-65735-5)
Supplement: Supplementary file 1 — Supplementary Information. [file 41598_2024_65735_MOESM1_ESM.docx]

Supplementary Figures

**Title: Delivery of CDNF by AAV-mediated gene transfer protects dopamine neurons and regulates ER stress and inflammation in an acute MPTP mouse model of Parkinson’s disease**

Jinhan Nam^1^, Christopher T. Richie^2^, Brandon K Harvey^2^, Merja H. Voutilainen^1*^

^1^ Division of Pharmacology and Pharmacotherapy, Faculty of Pharmacy, University of Helsinki, Helsinki, Finland. ^2^ Intramural Research Program, National Institute on Drug Abuse, Baltimore, MD USA

**Keywords** Parkinson’s disease (PD), 1-methyl-4-phenyl-1,2,3,6-tetrahydropyridine (MPTP), Cerebral dopamine neurotrophic factor (CDNF), Neuroinflammation, UPR, Microglia, Astrocyte

***Correspondence**

Corresponding Author: Merja Voutilainen, Ph.D. (Pharm.)

merja.h.voutilainen@helsinki.fi


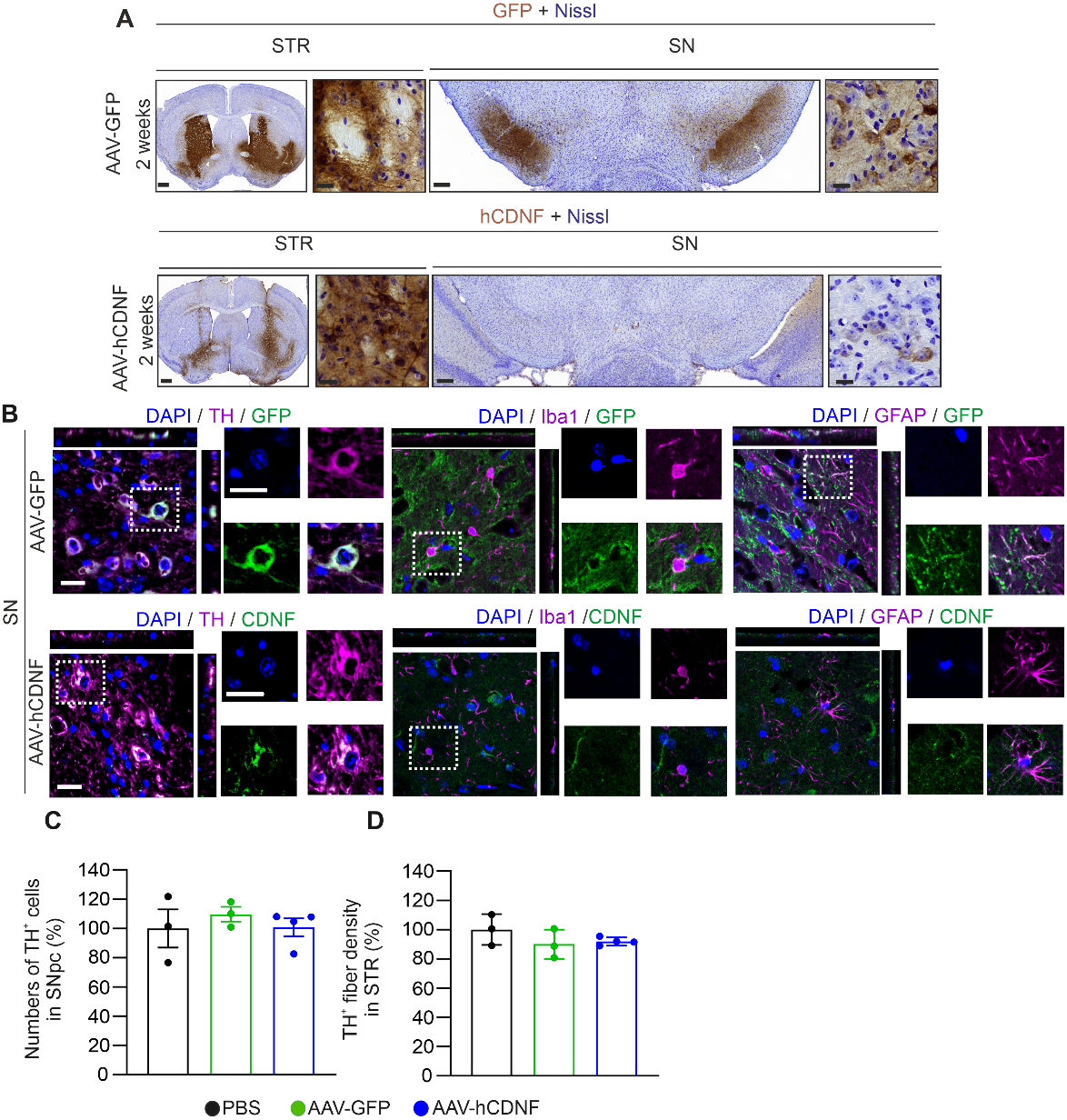


**Supplementary Figure 1. Characterization of AAV-GFP and AAV-hCDNF at 2 weeks after in intact mice.** AAV-GFP, AAV-hCDNF were bilaterally injected into the striatum in mice. And 2 weeks later, mice were sacrificed for immunostaining. ***A***, Immunohistochemical staining for STR and SN sections with GFP (upper, brown), CDNF (lower, brown), and counterstained with Cresyl violet (Nissl). Scale bar 500 µm for STR, 200 µm for SN, and 20 µm for enlarged images. ***B****,* Immunofluorescence of dopaminergic neurons, (TH; Magenta), microglia (Iba1; Magenta) and astrocyte (GFAP: Magenta) with GFP (Green) or hCDNF (Green) in the SN in 6weeks later AAV injection. Scale bar 20 µm. ***C***, Quantification for the numbers of TH-positive neurons in the SNpc. F (2, 7) = 0.3905, p = 0.6906. ***D***, Quantification for the density of TH^+^ fibers in the STR. F (2, 7) = 1.364, p = 0.3160. All data are expressed as Mean ± SEM. *n* = 3 for 2 weeks PBS, *n* = 3 for 2weeks AAV-GFP, *n* = 3 for 2weeks AAV-hCDNF. (**C**, **D**) One-way ANOVA was used, followed by Tukey’s post hoc test for multiple comparisons. All data are expressed as Mean ± S.E.M.


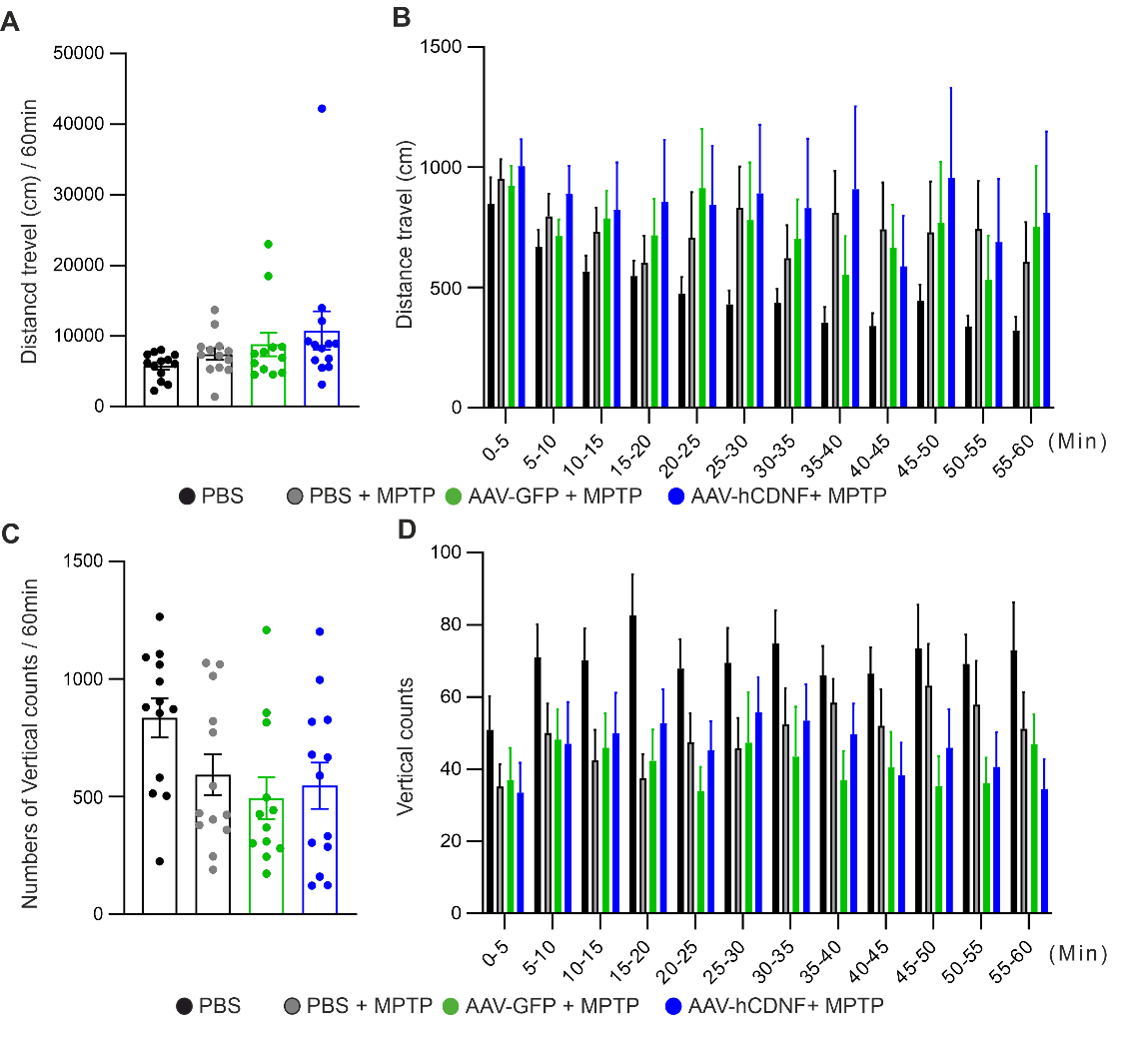


**Supplementary Figure 2. hCDNF did not affect travel distance and vertical counts in locomotor activity. A**, Total distance traveled for 60 min was recorded, F (3, 47) = 1.617, p = 0.1981. **B**, The distance traveled was recorded by a 5 min interval, F (33, 564) = 0.2401, p > 0.9999. **C**, Vertical counts (rearing-up) for 60 min were recorded, F (3, 47) = 2.844, p = 0.0477. **D**, Vertical counts (rearing-up) were recorded by a 5 min interval, F (33, 564) = 0.4685, p = 0.9954. n = 13 for PBS, n = 13 for PBS + MPTP, n = 12 for AAV-GFP + MPTP, and n = 13 for AAV-hCDNF. One-way (**A**, **C**) or two-way (**B**, **D**) ANOVA was used, followed by Tukey’s post hoc test for multiple comparisons. All data are expressed as Mean ± S.E.M.


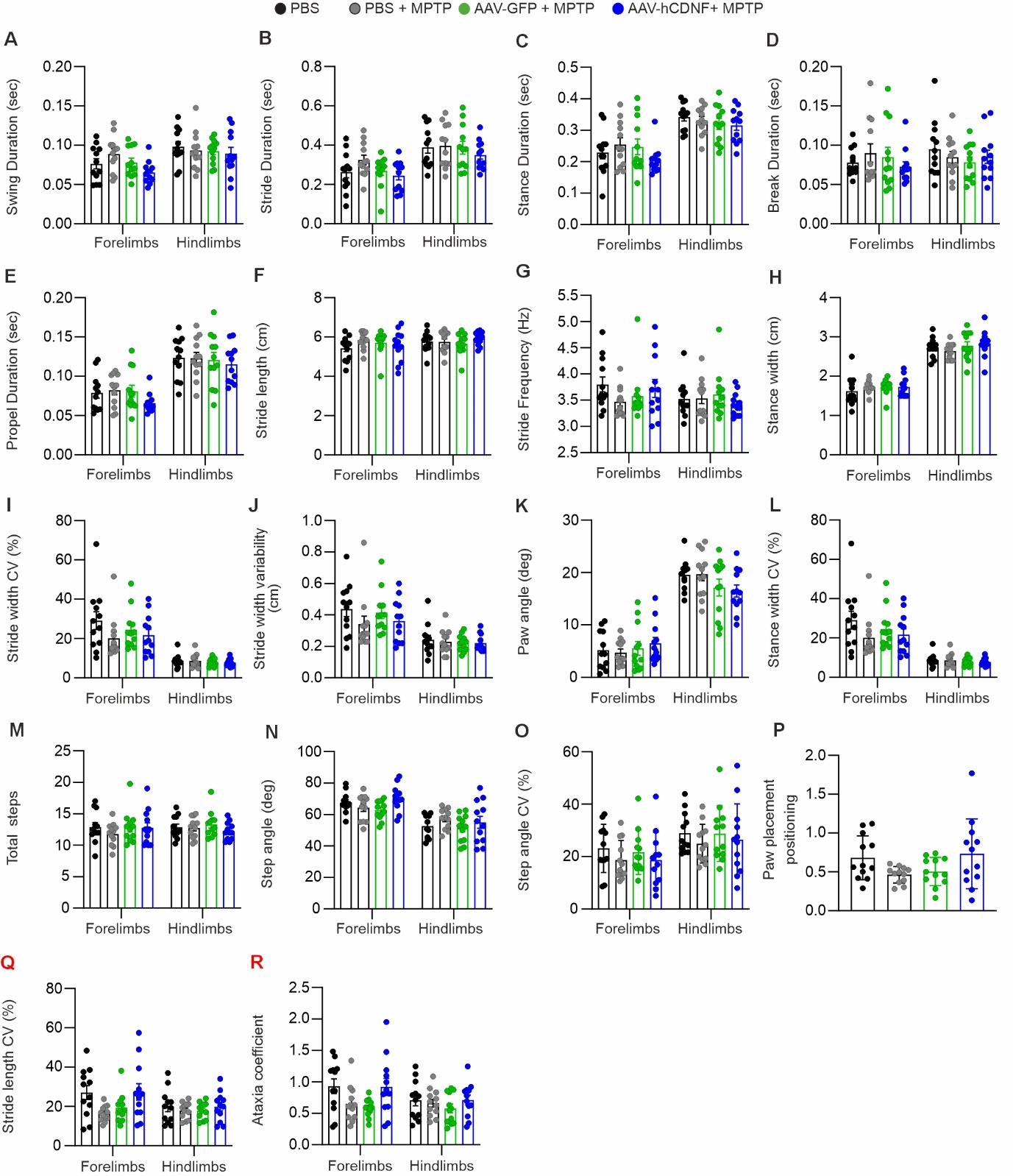


**Supplementary Figure 3. Gait dynamics in the AAV-hCDNF treated acute MPTP model.** Six days after the last injection of MPTP, mice gait dynamics were evaluated using the DigiGait apparatus. **A**, Swing duration. **B**, Stride duration. **C**, Stance duration. **D**, Break duration. **E**, Propel duration. **F**,. **G**, Stride Frequency. **H**, Stance width. **I**, Stride width Coefficient of Variation (CV. **J**, Stride width variability. **K**, Paw angle. **L**, Stance width CV. **M**, Total steps. **N**, Step angle. **O**, Step angle CV. **P**, Paw placement positioning, F(3, 44) = 2.553, p = 0.0676. **Q,** Stride length Coefficient of Variation (CV), and **R**, Ataxia coefficient.

n = 12 for PBS, n = 12 for PBS + MPTP, n = 12 for AAV-GFP + MPTP, and n = 12 for AAV-hCDNF. One-way (**P**) or two-way (**A**-**O**) ANOVA was used, followed by Tukey’s post hoc test for multiple comparisons. All data are expressed as Mean ± S.E.M.


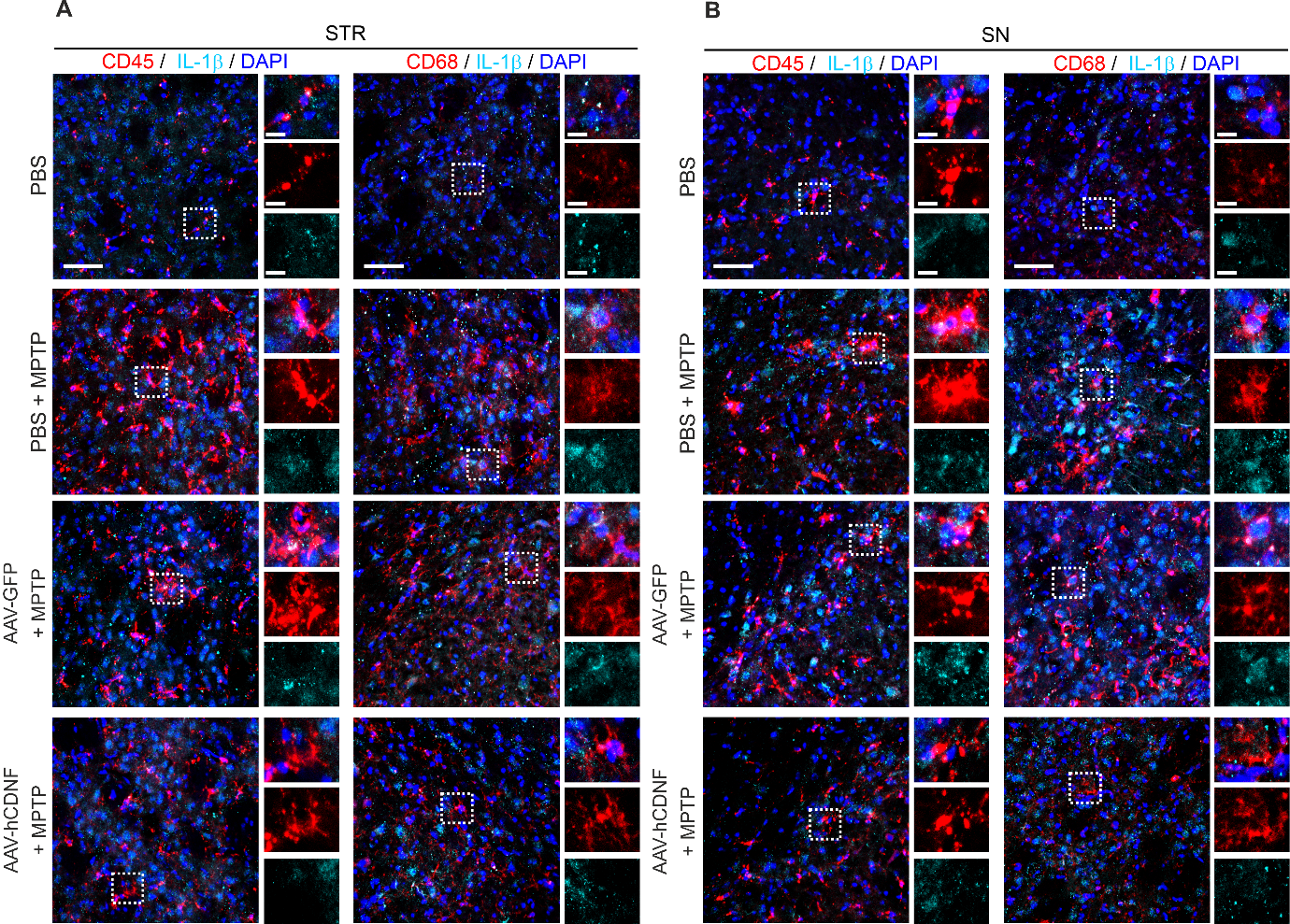


**Supplementary Figure 4. Double-immunofluorescence images for IL-1β with CD45 or CD68 in the STR and SN.** Representative images of double-immunofluorescence for DAPI (Blue), CD45 and CD68 (Red), and IL-1β (Cyan) in the STR (**A**) and SN (**B**). Dotted white rectangles indicates enlarged area. Scale bar 50 µm, 10 µm for enlarge.

**Table 1 Description of Gait Parameters**

| Index | Definition |
| --- | --- |
| Swing Duration (sec) | Time duration of paw complete no contact with belt. |
| Stride Duration (sec) | Time duration of one complete stride. (Stride duration = Swing duration + Stance duration). |
| Stance Duration (sec) | Time duration of paw complete contact with belt. |
| Brake Duration (sec) | Time duration of brake portion in stance phase. (From initial paw contact to complete paw contact |
| Propel Duration (sec) | Time duration of propel portion in stance phase. (From complete paw contact to nearly before swing phase) |
| Stride length (cm) | Distance between the strides for contacted one foot and opposite foot. |
| Stride Frequency (Hz) | Number of complete strides each second. |
| Stance Width (cm) | The distance between the centers of gravity of each set of pivot legs during points stance. |
| Stride Width CV (%) | Coefficient of variation of stride width. |
| Stride Width Variability (cm) | The standard deviation of the stride width for the set of strides recorded. |
| Paw angle (deg) | The angle paw makes with the long axis of the animal’s movement direction. |
| Stance Width CV (%) | Coefficient of variation of stance width. |
| Toal steps (real number) | The number of steps included in the analysis |
| Step angle (deg) | The angle between left and right hind paws as a function of stride length and stand width. |
| Step angle CV | Coefficient of variation of step angle. |
| Paw Placement Positioning (cm) | This is the amount of overlap between the ipsilateral forefoot and backfoot during full stance. |
| Stride length variability (cm) | The standard deviation of the stride length for the set of strides recorded |
| Stride length CV (%) | Coefficient of variation of stride length |
| Ataxia coefficient (real number) | An index of step-to-step variability |

**Table 2. Statistical tests used for data analysis**

| Figure | Statistical test | Descriptive statistics |
| --- | --- | --- |
| Fig1. H | Ordinary one-way ANOVA with  Tukey's multiple comparisons test | TH: F_(2, 13)_ = 1.143, p = 0.3489.  PBS vs. AAV-GFP, p = 0.9585  PBS vs. CDNF, p = 0.5177  AAV-GFP vs. CDNF, p = 0.3633  DAT: F_(2, 13)_ = 1.474, p= 0.2649.  PBS vs. AAV-GFP, p= 0.2400  PBS vs. CDNF, p=0.7526  AAV-GFP vs. CDNF, p=0.5568 |
| Fig1. J | Ordinary one-way ANOVA with  Tukey's multiple comparisons test | F_(2, 13)_ = 0.03687, p = 0.9639,  PBS vs. GFP, p = 0.9856  PBS vs. CDNF, p = 0.9944  GFP vs. CDNF, p = 0.9606 |
| Fig2. C | Ordinary one-way ANOVA with  Tukey's multiple comparisons test | F_(3, 47)_ = 15.93, p < 0.0001.  PBS vs. MPTP, p= <0.0001  PBS vs. AAV-GFP +MPTP, p = <0.0001  PBS vs. AAV-hCDNF + MPTP, p= 0.0457  MPTP vs. AAV-GFP +MPTP, p = 0.9994  MPTP vs. AAV-hCDNF + MPTP, p = 0.0151  AAV-GFP +MPTP vs. AAV-hCDNF + MPTP, p = 0.0130 |
| Fig2. D | Ordinary one-way ANOVA with  Tukey's multiple comparisons test | F_(3, 20)_ = 10.12, p = 0.0003.  PBS vs. MPTP, p = 0.0017  PBS vs. AAV-GFP +MPTP, p = 0.0519  PBS vs. AAV-hCDNF + MPTP, p = 0.9911  MPTP vs. AAV-GFP +MPTP, p = 0.6143  MPTP vs. AAV-hCDNF + MPTP, p = 0.0009  AAV-GFP +MPTP vs. AAV-hCDNF + MPTP, p = 0.0291 |
| Fig2. E | Ordinary one-way ANOVA with  Tukey's multiple comparisons test | F_(3, 20)_ = 2.191, p = 0.1208.  PBS vs. MPTP, p = 0.0933  PBS vs. AAV-GFP +MPTP, p = 0.7770  PBS vs. AAV-hCDNF + MPTP, p = 0.8704  MPTP vs. AAV-GFP +MPTP, p = 0.5286  MPTP vs. AAV-hCDNF + MPTP, p = 0.3529  AAV-GFP +MPTP vs. AAV-hCDNF + MPTP, p = 0.9958 |
| Fig2. F | Ordinary one-way ANOVA with  Tukey's multiple comparisons test | F_(3, 20)_ = 2.466, p = 0.0919.  PBS vs. MPTP, p = 0.1066  PBS vs. AAV-GFP +MPTP, p = 0.1407  PBS vs. AAV-hCDNF + MPTP, p = 0.3549  MPTP vs. AAV-GFP +MPTP, p >0.9999  MPTP vs. AAV-hCDNF + MPTP, p= 0.7524  AAV-GFP +MPTP vs. AAV-hCDNF + MPTP, p = 0.7722 |
| Fig. 2H | Ordinary one-way ANOVA with  Tukey's multiple comparisons test | Forelimbs: F_(3, 44)_ = 3.933, p = 0.0143  PBS vs. MPTP, p = 0.0509  PBS vs. AAV-GFP +MPTP, p = 0.2086  PBS vs. AAV-hCDNF + MPTP, p = 0.9995  MPTP vs. AAV-GFP +MPTP, p = 0.9056  MPTP vs. AAV-hCDNF + MPTP, p= 0.0390  AAV-GFP +MPTP vs. AAV-hCDNF + MPTP, p = 0.1699  Hindlimbs: F_(3, 44)_ = 1.481, p = 0.2328  PBS vs. MPTP, p = 0.9011  PBS vs. AAV-GFP +MPTP, p = 0.3712  PBS vs. AAV-hCDNF + MPTP, p = 0.9897  MPTP vs. AAV-GFP +MPTP, p = 0.7810  MPTP vs. AAV-hCDNF + MPTP, p = 0.7524  AAV-GFP +MPTP vs. AAV-hCDNF + MPTP, p = 0.2263 |
| Fig. 3B | Ordinary one-way ANOVA with  Tukey's multiple comparisons test | F_(3, 24)_ = 50.4 p < 0.0001.  PBS vs. MPTP, p < 0.0001  PBS vs. AAV-GFP +MPTP, p < 0.0001  PBS vs. AAV-hCDNF + MPTP, p < 0.0001  MPTP vs. AAV-GFP +MPTP, p = 0.3194  MPTP vs. AAV-hCDNF + MPTP, p = 0.0008  AAV-GFP +MPTP vs. AAV-hCDNF + MPTP, p = 0.0511 |
| Fig. 3C | Ordinary one-way ANOVA with  Tukey's multiple comparisons test | F_(3, 24)_ = 90.3, p < 0.0001.  PBS vs. MPTP, p < 0.0001  PBS vs. AAV-GFP +MPTP, p < 0.0001  PBS vs. AAV-hCDNF + MPTP, p < 0.0001  MPTP vs. AAV-GFP +MPTP, p > 0.9999  MPTP vs. AAV-hCDNF + MPTP, p = 0.0110  AAV-GFP +MPTP vs. AAV-hCDNF + MPTP, p = 0.0111 |
| Fig. 3D | Ordinary one-way ANOVA with  Tukey's multiple comparisons test  And  Unpaired Two-tailed *t* test | F_(3, 24)_ = 52.1, p < 0.0001.  PBS vs. MPTP, p < 0.0001  PBS vs. AAV-GFP +MPTP, p < 0.0001  PBS vs. AAV-hCDNF + MPTP, p<0.0001  MPTP vs. AAV-GFP +MPTP, p = 0.6139  MPTP vs. AAV-hCDNF + MPTP, p = 0.009  AAV-GFP +MPTP vs. AAV-hCDNF + MPTP, p = 0.1307  Unpaired Two-tailed *t* test  AAV-GFP +MPTP vs. AAV-hCDNF + MPTP, t_(1.999)_ = 12, p = 0.0688 |
| Fig. 3F | Ordinary one-way ANOVA with  Tukey's multiple comparisons test | F_(3, 19)_ = 59.41, p < 0.0001.  PBS vs. MPTP, p < 0.0001  PBS vs. AAV-GFP +MPTP, p < 0.0001  PBS vs. AAV-hCDNF + MPTP, p < 0.0001  MPTP vs. AAV-GFP +MPTP, p = 0.5616  MPTP vs. AAV-hCDNF + MPTP, p = 0.0002  AAV-GFP +MPTP vs. AAV-hCDNF + MPTP, p = 0.0069 |
| Fig. 3G | Ordinary one-way ANOVA with  Tukey's multiple comparisons test | F_(3, 19)_ = 23.02, p < 0.0001.  PBS vs. MPTP, p < 0.0001  PBS vs. AAV-GFP +MPTP, p <0 .0001  PBS vs. AAV-hCDNF + MPTP, p = 0.0089  MPTP vs. AAV-GFP +MPTP, p =0 .9865  MPTP vs. AAV-hCDNF + MPTP, p= 0.0137  AAV-GFP +MPTP vs. AAV-hCDNF + MPTP, p= 0.0093 |
| Fig4. C | Ordinary one-way ANOVA with  Tukey's multiple comparisons test | STR: F_(3, 12)_ = 8.815, p = 0.0023,  PBS vs. MPTP, p = 0.0290  PBS vs. AAV-GFP +MPTP, p = 0.0198  PBS vs. AAV-hCDNF + MPTP, p = 0.9672  MPTP vs. AAV-GFP +MPTP, p = 0.9962  MPTP vs. AAV-hCDNF + MPTP, p = 0.0137  AAV-GFP +MPTP vs. AAV-hCDNF + MPTP, p = 0.0089  SN: F_(3, 12)_ = 10.95, p = 0.0009.  PBS vs. MPTP, p = 0.0102  PBS vs. AAV-GFP +MPTP, p = 0.0098  PBS vs. AAV-hCDNF + MPTP, p= 0.9885  MPTP vs. AAV-GFP +MPTP, p>0.9999  MPTP vs. AAV-hCDNF + MPTP, p= 0.0059  AAV-GFP +MPTP vs. AAV-hCDNF + MPTP, p= 0.0057 |
| Fig4. E | Ordinary one-way ANOVA with  Tukey's multiple comparisons test | Pro-IL1β: F_(2, 14)_ = 9.257, p = 0.0027.  PBS vs. AAV-GFP +MPTP, p = 0.0023  PBS vs. AAV-hCDNF + MPTP, p = 0.4235  AAV-GFP +MPTP vs. AAV-hCDNF + MPTP, p = 0.0246  Active-IL1β: F_(3, 19)_ = 8.8892, p = 0.0007.  PBS vs. MPTP, p = 0.0269  PBS vs. AAV-GFP +MPTP, p = 0.0120  PBS vs. AAV-hCDNF + MPTP, p = 0.9154  MPTP vs. AAV-GFP +MPTP, p =9526  MPTP vs. AAV-hCDNF + MPTP, p= 0.0067  AAV-GFP +MPTP vs. AAV-hCDNF + MPTP, p = 0.0031 |
| Fig4. F | Ordinary one-way ANOVA with  Tukey's multiple comparisons test | Pro-IL1β : F_(2, 14)_ = 30.30, p < 0.0001.  PBS vs. AAV-GFP +MPTP, p = 0.0126  PBS vs. AAV-hCDNF + MPTP, p = 0.0012  AAV-GFP +MPTP vs. AAV-hCDNF + MPTP, p < 0.0001  Active-IL1β : F_(2, 14)_ = 3.540, p = 0.0570.  PBS vs. AAV-GFP +MPTP, p = 0.2057  PBS vs. AAV-hCDNF + MPTP, p = 0.6699  AAV-GFP +MPTP vs. AAV-hCDNF + MPTP, p = 0.0493 |
| Fig4. I | Ordinary one-way ANOVA with  Tukey's multiple comparisons test | F_(3, 12)_ = 30.89, p < 0.0001.  PBS vs. MPTP, p < 0.0001  PBS vs. AAV-GFP +MPTP, p = 0.0005  PBS vs. AAV-hCDNF + MPTP, p = 0.9989  MPTP vs. AAV-GFP +MPTP, p = 0.3306  MPTP vs. AAV-hCDNF + MPTP, p < 0.0001  AAV-GFP +MPTP vs. AAV-hCDNF + MPTP, p = 0.0004 |
| Fig4 J | Ordinary one-way ANOVA with  Tukey's multiple comparisons test | F_(3, 12)_ = 18.76, p < 0.0001.  PBS vs. MPTP, p = 0.0004  PBS vs. AAV-GFP +MPTP, p = 0.0005  PBS vs. AAV-hCDNF + MPTP, p = 0.7172  MPTP vs. AAV-GFP +MPTP, p = 0.9946  MPTP vs. AAV-hCDNF + MPTP, p = 0.0020  AAV-GFP +MPTP vs. AAV-hCDNF + MPTP, p = 0.0030 |
| Fig5. C | Ordinary one-way ANOVA with  Tukey's multiple comparisons test | STR: F_(3, 12)_ = 14.66, p = 0.0003,.  PBS vs. MPTP, p = 0.0170  PBS vs. AAV-GFP +MPTP, p = 0.0005  PBS vs. AAV-hCDNF + MPTP, p = 0.9552  MPTP vs. AAV-GFP +MPTP, p = 0.1870  MPTP vs. AAV-hCDNF + MPTP, p = 0.0415  AAV-GFP +MPTP vs. AAV-hCDNF + MPTP, p = 0.0010  SN: F_(3, 12)_ = 23.48, p < 0.0001.  PBS vs. MPTP, p = 0.0002  PBS vs. AAV-GFP +MPTP, p = 0.0002  PBS vs. AAV-hCDNF + MPTP, p = 0.7411  MPTP vs. AAV-GFP +MPTP, p > .9999  MPTP vs. AAV-hCDNF + MPTP, p = 0.0008  AAV-GFP +MPTP vs. AAV-hCDNF + MPTP, p = 0.0008 |
| Fig5. F | Ordinary one-way ANOVA with  Tukey's multiple comparisons test | STR: F_(3, 12)_ = 40.64, p < 0.0001,  PBS vs. MPTP, p < 0.0001  PBS vs. AAV-GFP +MPTP, p < 0.0001  PBS vs. AAV-hCDNF + MPTP, p = 0.0028  MPTP vs. AAV-GFP +MPTP, p = 0.9984  MPTP vs. AAV-hCDNF + MPTP, p = 0.0026  AAV-GFP +MPTP vs. AAV-hCDNF + MPTP, p = 0.0020  SN: F_(3, 12)_ = 17.44, p = 0.0001).  PBS vs. MPTP, p = 0.0006  PBS vs. AAV-GFP +MPTP, p = 0.0003  PBS vs. AAV-hCDNF + MPTP, p = 0.3157  MPTP vs. AAV-GFP +MPTP, p = 0.9716  MPTP vs. AAV-hCDNF + MPTP, p = 0.0113  AAV-GFP +MPTP vs. AAV-hCDNF + MPTP, p = 0.0053 |
| Fig6. C | Ordinary one-way ANOVA with  Tukey's multiple comparisons test | STR: F_(3, 12)_ = 15.69, p = 0.0002,  PBS vs. MPTP, p= 0.0052  PBS vs. AAV-GFP +MPTP, p = 0.0005  PBS vs. AAV-hCDNF + MPTP, p = 0.9310  MPTP vs. AAV-GFP +MPTP, p = 0.4750  MPTP vs. AAV-hCDNF + MPTP, p = 0.0146  AAV-GFP +MPTP vs. AAV-hCDNF + MPTP, p = 0.0012  SN: F_(3, 12)_ = 12.33, p = 0.0006.  PBS vs. MPTP, p= 0.0074  PBS vs. AAV-GFP +MPTP, p= 0.0052  PBS vs. AAV-hCDNF + MPTP, p= 0.9937  MPTP vs. AAV-GFP +MPTP, p=0.9970  MPTP vs. AAV-hCDNF + MPTP, p = 0.0047  AAV-GFP +MPTP vs. AAV-hCDNF + MPTP, p= 0.0034 |
| Fig6. D | Ordinary one-way ANOVA with  Tukey's multiple comparisons test  And  Unpaired Two-tailed *t* test | F_(3, 28)_ = 5.31, p = 0.0050.  PBS vs. MPTP, p = 0.1663  PBS vs. AAV-GFP +MPTP, p = 0.0062  PBS vs. AAV-hCDNF + MPTP, p = 0.5291  MPTP vs. AAV-GFP +MPTP, p = 0.1843  MPTP vs. AAV-hCDNF + MPTP, p = 0.09247  AAV-GFP +MPTP vs. AAV-hCDNF + MPTP, p = 0.0836    Unpaired Two-tailed *t* test  PBS vs. MPTP*, t_(_*_4.695)_ = 14.82, p = 0.0003 |
| Fig6. E | Ordinary one-way ANOVA with  Tukey's multiple comparisons test | F_(3, 28)_ = 0.666, p = 0.5798.  PBS vs. MPTP, p= 0.6580  PBS vs. AAV-GFP +MPTP, p= 0.9791  PBS vs. AAV-hCDNF + MPTP, p= 0.9995  MPTP vs. AAV-GFP +MPTP, p=0.8954  MPTP vs. AAV-hCDNF + MPTP, p = 0.6140  AAV-GFP +MPTP vs. AAV-hCDNF + MPTP, p= 0.9616 |
| Fig6. G | Ordinary one-way ANOVA with  Tukey's multiple comparisons test | STR: F_(3, 12)_ = 10.87, p = 0.001,  PBS vs. MPTP, p= 0.0037  PBS vs. AAV-GFP +MPTP, p= 0.0030  PBS vs. AAV-hCDNF + MPTP, p= 0.6641  MPTP vs. AAV-GFP +MPTP, p=0.9990  MPTP vs. AAV-hCDNF + MPTP, p = 0.0276  AAV-GFP +MPTP vs. AAV-hCDNF + MPTP, p= 0.0218  SN: F_(3, 12)_ = 13.65, p = 0.0004.  PBS vs. MPTP, p= 0.0018  PBS vs. AAV-GFP +MPTP, p= 0.0393  PBS vs. AAV-hCDNF + MPTP, p= 0.9248  MPTP vs. AAV-GFP +MPTP, p=0.3176  MPTP vs. AAV-hCDNF + MPTP, p = 0.0006  AAV-GFP +MPTP vs. AAV-hCDNF + MPTP, p= 0.0133 |
| Fig6. I | Ordinary one-way ANOVA with  Tukey's multiple comparisons test  And  Unpaired Two-tailed *t* test | F_(3, 28)_ = 3.004, p = 0.0471.  PBS vs. MPTP, p = 0.3526  PBS vs. AAV-GFP +MPTP, p= 0.0300  PBS vs. AAV-hCDNF + MPTP, p = 0.7232  MPTP vs. AAV-GFP +MPTP, p = 0.4577  MPTP vs. AAV-hCDNF + MPTP, p = 0.6545  AAV-GFP +MPTP vs. AAV-hCDNF + MPTP, p = 0.2730  Unpaired Two-tailed *t* test  PBS vs. MPTP, *t*_(1.914)_ = 15.19, p = 0.0747. |
| Fig6. J | Ordinary one-way ANOVA with  Tukey's multiple comparisons test  And  Unpaired Two-tailed *t* test | F_(3, 28)_ = 2.36, p = 0.0926.  PBS vs. MPTP, p = 0.1168  PBS vs. AAV-GFP +MPTP, p = 0.7276  PBS vs. AAV-hCDNF + MPTP, p = 0.9998  MPTP vs. AAV-GFP +MPTP, p = 0.6662  MPTP vs. AAV-hCDNF + MPTP, p = 0.1610  AAV-GFP +MPTP vs. AAV-hCDNF + MPTP, p = 0.7881  Unpaired Two-tailed *t* test  PBS vs. MPTP, *t*_(1.904)_ = 16, p = 0.0751. |


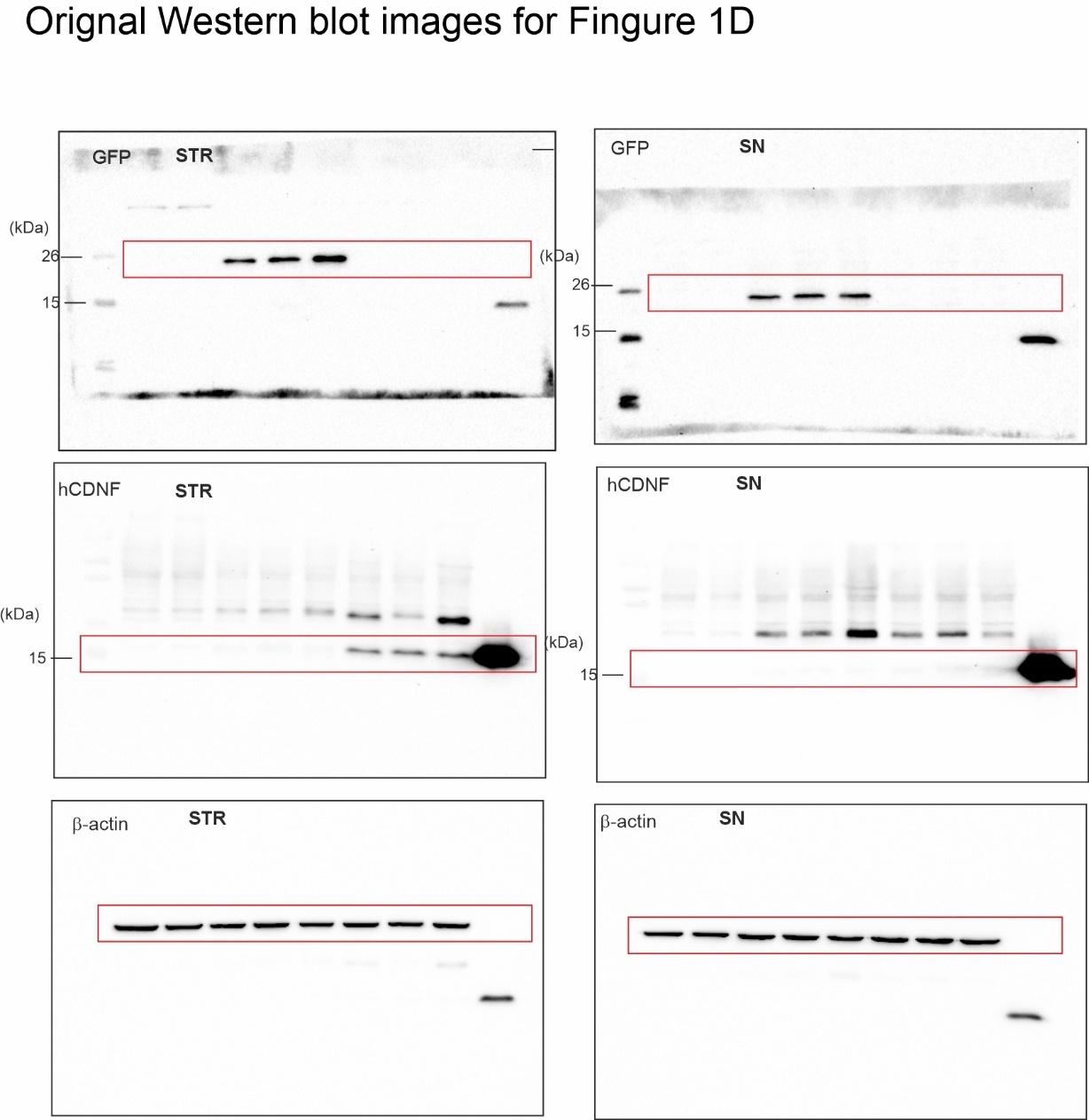


**Supplementary Figure 5. Original immunoblots for Figure1 D.** Cropped areas were represented by red rectangles. Using stripping buffer, immunoblots were sequentially analyzed with multiple antibodies. Antibodies are subsequent to hCDNF, GFP, and β-actin.


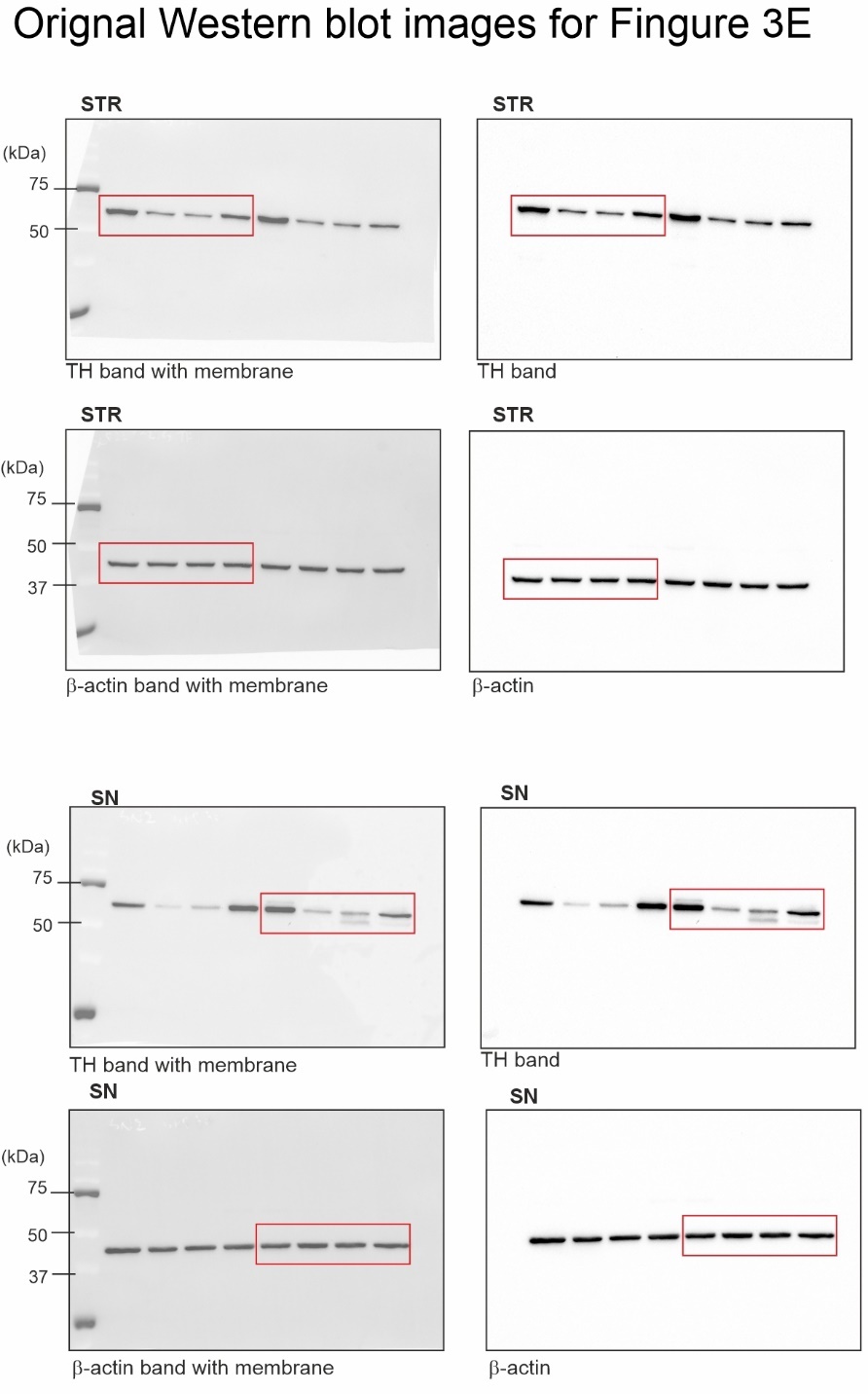


**Supplementary Figure 6. Original immunoblots for Figure3 E.** Cropped areas were represented by red rectangles. Using stripping buffer, immunoblots were sequentially analyzed with multiple antibodies. Antibodies are subsequent to TH, β-actin.


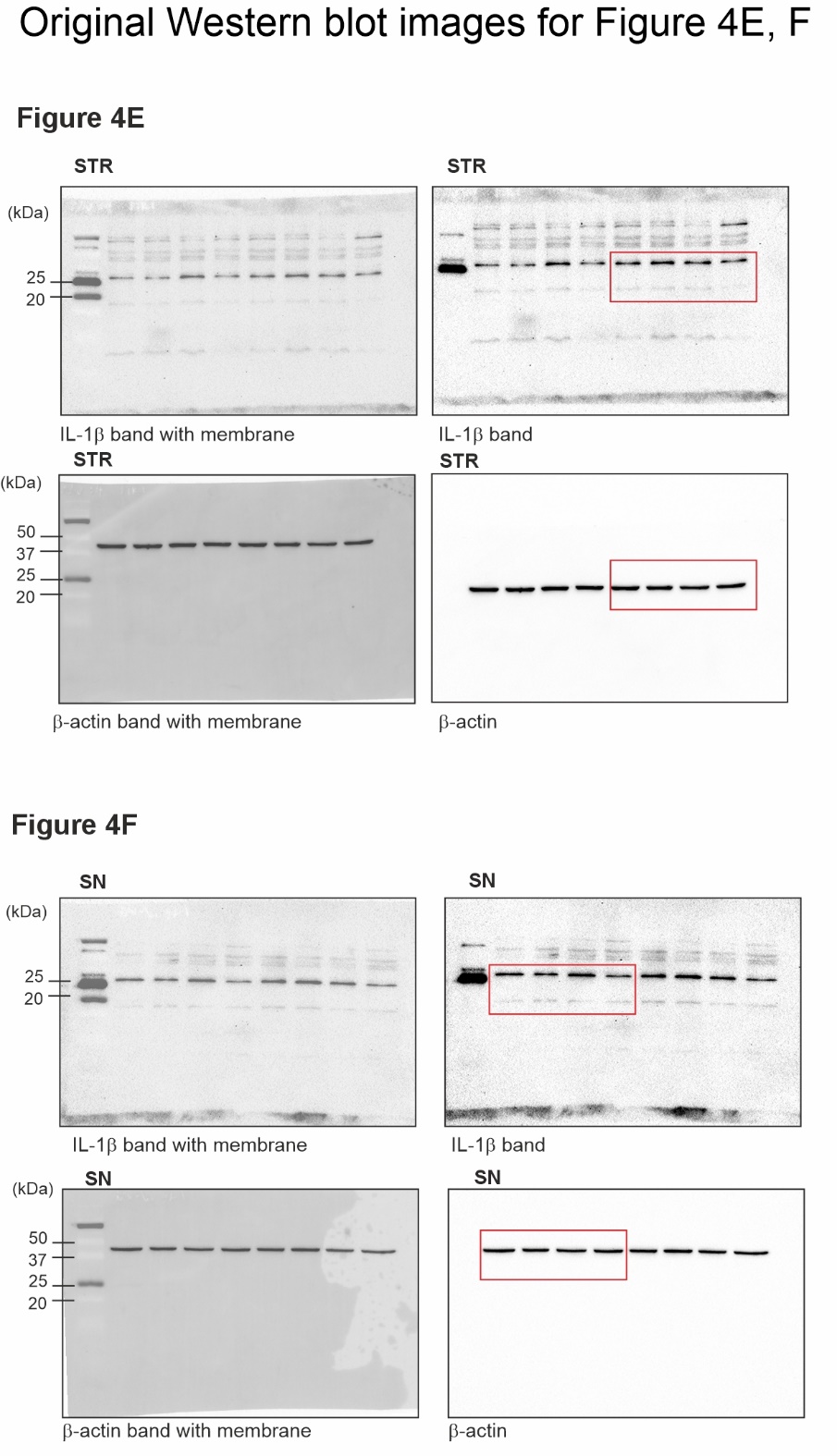


**Supplementary Figure 7. Original immunoblots for Figure4 E, F.** Cropped areas were represented by red rectangles. Using stripping buffer, immunoblots were sequentially analyzed with multiple antibodies. Antibodies are subsequent to IL-1β, β-actin.


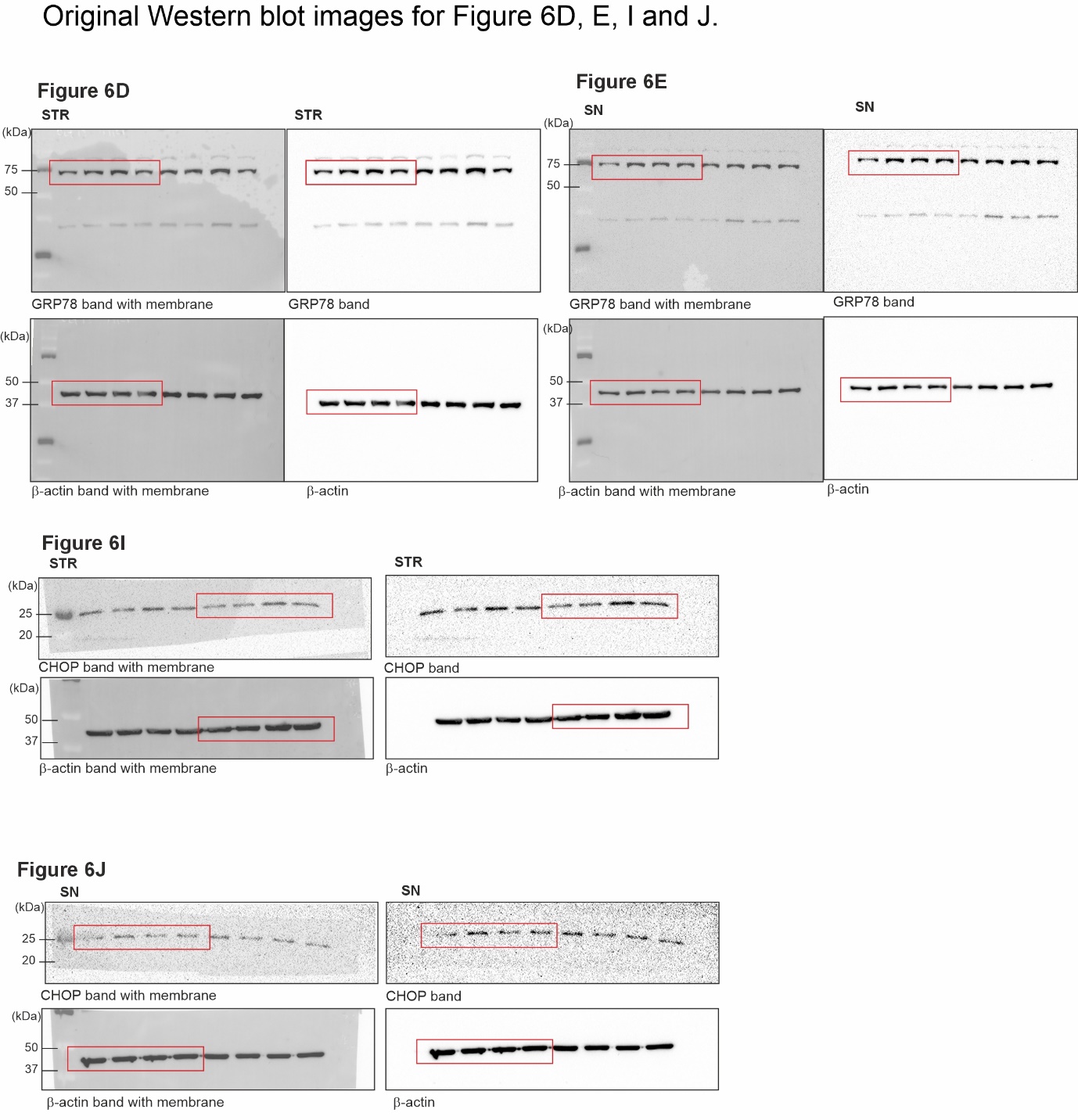


**Supplementary Figure 8. Original immunoblots for Figure6 D, E, I and J.** Cropped areas were represented by red rectangles. Using stripping buffer, immunoblots were sequentially analyzed with multiple antibodies. Antibodies are subsequent to GRP78, CHOP, and β-actin.
